# Alcohol, empathy, and morality: acute effects of alcohol consumption on affective empathy and moral decision-making

**DOI:** 10.1007/s00213-019-05314-z

**Published:** 2019-07-09

**Authors:** Kathryn B. Francis, Michaela Gummerum, Giorgio Ganis, Ian S. Howard, Sylvia Terbeck

**Affiliations:** 1 School of Psychology, University of Plymouth, Drake Circus, Plymouth, PL4 8AA UK; 2 Present Address: Division of Psychology, Faculty of Management, Law & Social Sciences, University of Bradford, Bradford, West Yorkshire BD7 1DP UK; 3 Centre for Robotics and Neural Systems, University of Plymouth, Drake Circus, Plymouth, PL4 8AA UK

**Keywords:** Moral decision-making, Moral judgment, Moral action, Alcohol, Empathy, Virtual reality

## Abstract

**Rationale:**

Hypothetical moral dilemmas, pitting characteristically utilitarian and non-utilitarian outcomes against each other, have played a central role in investigations of moral decision-making. Preferences for utilitarian over non-utilitarian responses have been explained by two contrasting hypotheses; one implicating increased deliberative reasoning, and the other implicating diminished harm aversion. In recent field experiments, these hypotheses have been investigated using alcohol intoxication to impair both social and cognitive functioning. These studies have found increased utilitarian responding, arguably as a result of alcohol impairing affective empathy.

**Objectives:**

The present research expands existing investigations by examining the acute effects of alcohol on affective empathy and subsequent moral judgments in traditional vignettes and moral actions in virtual reality, as well as physiological responses in moral dilemmas.

**Methods:**

Participants (*N* = 48) were administered either a placebo or alcohol in one of two dosages; low or moderate. Both pre- and post intervention, participants completed a moral action and moral judgment task alongside behavioural measures of affective empathy.

**Results:**

Higher dosages of alcohol consumption resulted in inappropriate empathic responses to facial displays of emotion, mirroring responses of individuals high in trait psychopathy, but empathy for pain was unaffected. Whilst affective empathy was influenced by alcohol consumption in a facial responding task, both moral judgments and moral actions were unaffected.

**Conclusions:**

These results suggest that facets, beyond or in addition to deficits in affective empathy, might influence the relationship between alcohol consumption and utilitarian endorsements.

**Electronic supplementary material:**

The online version of this article (10.1007/s00213-019-05314-z) contains supplementary material, which is available to authorized users.

## Introduction

Traditionally, provocative moral dilemmas, pitting characteristically utilitarian versus deontological ideologies against each other, have played a central role in the investigation of moral judgment (e.g. Bartels et al. 2015; Cushman et al. 2010). For example, in the *switch* dilemma, individuals must decide whether to flick a switch, redirecting a trolley car to kill one worker on the tracks instead of five (Thomson 1976). Alternatively, in the *footbridge* dilemma, individuals must decide whether to push a large person in front of the trolley, in order to stop it from killing the five workers on the tracks (Foot 1978). This ‘trolley problem’ has generated interest as individuals tend to give the typically consequentialist or utilitarian judgment (they judge that maximising the number of lives saved is morally acceptable) in the *switch* case, but refuse to do so in a characteristically deontological sense (harm is wrong and the ends do not justify the means) in the *footbridge* case (Thomson 1976).

Several theories have attempted to understand these divergent responses given their structural similarity in entailing the five-for-one trade-off (Thomson 1976). Greene’s dual process model of moral judgment (Greene et al. 2001) distinguishes between ‘personal’ dilemmas like the *footbridge* and ‘impersonal’ dilemmas such as the *switch*. Personal dilemmas are those ‘involving actions that are (a) likely to cause serious bodily harm, (b) to a particular person, where (c) this harm does not result from deflecting an existing threat onto a different party’ (Greene et al. 2001, p. 2107). These dilemmas are thought to trigger an emotional and aversive response; an ‘alarm bell’ associated with emotional systems in the brain (Cushman et al. 2010, p. 50) resulting in a deontological or non-utilitarian response (i.e. refusing to endorse harmful actions). In the absence of this negative alarm bell in impersonal dilemmas, the utilitarian option dominates the response, driven by increased activations in brain areas associated with controlled cognitive processes (Greene et al. 2001).

Greene’s model has received attention across many research domains, with a strong body of research supporting the theory that deliberative reasoning results in greater utilitarian moral judgments (e.g. Greene et al. 2008; Greene et al. 2004; Greene et al. 2001; Koenigs et al. 2007). However, the proposed link between utilitarian responses and increased reasoning has also been challenged (e.g. Kahane et al. 2015) with research suggesting that increased utilitarian preference may, instead, derive from a decreased aversion to harming others as a result of deficits in social processing (Patil 2015). For example, research suggests that the increased ‘utilitarianism’ found in psychopathic populations, results from deficits in affective empathy^1^ (Bartels and Pizarro 2011; Djeriouat and Tremoliere 2014; Gao and Tang 2013). In fact, recent investigations of moral actions in virtual reality trolley problems have found a positive association between psychopathic traits and utilitarian actions (Francis et al. 2016) and the strength of these actions (Francis et al. 2017b). These actions were also negatively associated with traits inversely related to psychopathy such as Honesty-Humility (sincerity, fairness, greed avoidance, and modesty). Support for this social processing hypothesis can also be found in pharmacological studies; Citalopram (a drug that enhances serotonin and subsequent pro-social behaviour) enhanced non-utilitarian moral responses (Crockett et al. 2010), whilst increased levels of testosterone (Carney and Mason 2010) and increased levels of anger (e.g. Choe and Min 2011) have been associated with increased utilitarian responses.

One way in which research has sought to investigate the relationship between social and cognitive functioning in moral decision-making is to study populations impaired in affective empathy and higher-order cognitive abilities. Previous research has examined individuals with deficits in emotional processing either as a result of brain lesions (e.g. Ciaramelli et al. 2007), neurological disorders (e.g. Mendez et al. 2005), or alcohol and drug dependence (Carmona-Perera et al. 2014; Carmona-Perera et al. 2013; Khemiri et al. 2012). To date, there have been few investigations of the acute effects of alcohol on moral judgments made in response to hypothetical moral dilemmas. In a recent study, Duke and Begue (2015) examined acute effects of blood alcohol concentration (BAC) on moral decision-making in participants recruited at bars in France. Across two studies, they found that BAC levels were positively correlated with utilitarian preferences in response to the *footbridge* dilemma and that this effect was not mediated by self-reported feelings of behavioural disinhibition or self-reported positive mood.

The finding that acute alcohol consumption promotes utilitarian moral judgments in response to personal moral dilemmas supports previous research examining moral decision-making in alcohol-dependent individuals (Carmona-Perera et al. 2014; Khemiri et al. 2012). These studies have found that prolonged effects of alcohol dependence result in greater utilitarian moral judgments as a result of affective processing deficits.^2^ Crucially, these findings are in contention with Greene’s dual process model which would argue that alcohol intoxication triggers emotional reactivity and impaired higher-order functioning, which in turn prompts increased non-utilitarian moral judgments (Greene et al. 2001). Duke and Begue (2015) argue that, instead, their finding implicates a strong role for impaired social cognition in predicting utilitarian preferences. Alcohol intoxication results in a ‘ … decreased capacity for empathy’ or more specifically, decreased aversion to harming others which subsequently promotes the utilitarian option (Duke and Begue 2015, p. 125). However, affective empathy was not explicitly measured in this paradigm. From a broader perspective, the theory that alcohol intoxication produces utilitarian responses as a result of impaired affective empathy is consistent with the connection between utilitarianism and certain deficits in social functioning as a result of brain damage (e.g. Koenigs et al. 2007) and psychopathic traits (e.g. Patil 2015).

Few studies have investigated both cognitive and affective empathy in alcohol-dependent individuals specifically (Thoma et al. 2013). Of the few that have examined this, some argue that impairments in premorbid trait empathy compromise social functioning, leading to more social problems, which could then predispose individuals to use alcohol as a coping strategy (Thoma et al. 2013). In terms of distinctions between affective and cognitive empathy in these individuals, several studies have found that affective empathy is principally impaired in alcohol-dependent individuals (Maurage et al. 2011; Marinkovic et al. 2009) and addicted patients more broadly (Ferrari et al. 2014). Whilst recent research has found that acute alcohol consumption reduces empathic neural activity for pain (Hu et al. 2017), the specific effects of acute alcohol intake on affective empathy have yet to be investigated. This is particularly important following suggestions that deficits in affective empathy mediate the relationship between alcohol consumption and utilitarian moral decision-making (Duke and Begue 2015).

## Addressing limitations

The quasi-experimental setup adopted by Duke and Begue (2015) raises questions regarding the influence of social atmosphere, potential social awareness, and uncontrolled alcohol dosages on moral decision-making. The present experiment addressed these limitations through a laboratory-controlled study, examining the effects of low and moderate dosages of alcohol consumption on moral judgments and moral actions. Whilst the research by Duke and Begue (2015) may have shed further light on the role of social deficits on utilitarian moral judgments, research has yet to investigate similar manipulations within the domain of moral action. This is significant given evidence of a disparity between moral judgments given in response to text-based vignettes and moral actions simulated in virtual trolley problems (e.g. Francis et al. 2016; Francis et al. 2017b; Francis et al. 2017a; McDonald et al. 2017; Patil et al. 2014). With previous research in this area supporting an association between moral actions and personality traits associated with diminished harm aversion (e.g. Francis et al. 2016; Francis et al. 2017b; Tassy et al. 2013a), exploring the effects of a diminished capacity to process social cues seems highly relevant in the domain of moral action. The present experiment was also adapted to include behavioural assessments of affective empathy and harm aversion in an attempt to shed light on the relationship between these traits and moral decision-making, beyond that of questionnaire assessments which can be confounded by social desirability responding.

## Specific hypotheses

### Moral responses

If existing research examining acute effects of alcohol (Duke and Begue 2015) and alcohol dependence (Carmona-Perera et al. 2014) on moral judgments in personal moral dilemmas (such as the *footbridge* dilemma) is supported, increased utilitarian preferences may be observed. If this relationship is influenced by deficits in social processing and reduced aversion to harm, performance in behavioural affective empathy tasks should decrease following alcohol consumption and this decline should be associated with utilitarian preferences in response to well-known moral vignettes (taken from Greene et al. (2001)). If, on the other hand, existing dual process models of moral judgment can be supported, then alcohol consumption might lead to increased preference for non-utilitarian moral judgments as a result of increased emotional reactivity and decreased cognitive functioning (Greene et al. 2001).

Predictions regarding simulated moral actions in a virtual reality version of the *footbridge* dilemma (used in Francis et al. (2016)) are less certain. Given previous research demonstrating the link between utilitarian moral actions and traits associated with reduced affective empathy and less aversion to harm (e.g. Francis et al. 2016; Francis et al. 2017b; Patil 2015; Tassy et al. 2013a), alcohol intoxication may result in greater utilitarian actions if affective empathy is diminished in behavioural measures of affective empathy. However, the latter hypothesis supporting Greene’s dual process model may also stand if existing models apply to the domain of moral action (e.g. Navarrete et al. 2012). In the present investigation, heart rate sampling was completed with the primary aims of assessing whether arousal was modality or moral specific (as in Francis et al. 2016) and, in this experiment specifically, to determine whether blood alcohol level affected physiological arousal in response to moral scenarios.

### Affective empathy

Self-reported valence (i.e. attraction or aversion) towards facial displays of emotion has been used as a measure of affective empathy (Wai and Tiliopoulos 2012). In the present investigation, personality traits were assessed to investigate their relationship with these behavioural measures of affective empathy, allowing validation of these behavioural approaches (Wai and Tiliopoulos 2012). If behavioural assessments provide a valid measure of trait affective empathy, performance in them is expected to inversely relate to primary psychopathy, as shown in previous research (Wai and Tiliopoulos 2012).^3^ In contrast, performance should positively correlate with existing trait assessments of affective empathy including Empathic Concern, a facet of the Interpersonal Reactivity Index (IRI) (Davis 1983), one of the longest used measures of empathy. Both Honesty-Humility, a trait relevant to reciprocal altruism (Ashton et al. 2014) thought to capture elements of empathic concern (Brick and Lewis 2016), as well as trait assessments of moral identity, should be positively associated with these behavioural assessments given that affective empathy, Honesty-Humility, and moral identity (internalisation) are thought to be connected facets of moral character relating to the consideration of others (Cohen et al. 2014; Cohen and Panter 2015).^4^ Previous research has also adopted empathy for pain tasks as a means of assessing affective responses to the pain of others (Decety and Jackson 2004; Jackson et al. 2005). If these provide a valid assessment of empathy for pain, reduced empathy for harm might be observed in individuals scoring higher in psychopathy and associated traits based on previous research (Bartels and Pizarro 2011; Patil 2015).

The second purpose of these behavioural affective empathy tasks was to provide a baseline with which to compare performances in post-intervention empathy tasks following alcohol consumption. If acute alcohol intake does affect social processing, by reducing the capacity for affective empathy and decreasing harm aversion, then alcohol consumption is expected to reduce performance in these behavioural measures of affective empathy. Self-reported valence towards happy and sad faces and self-reported empathy towards painful images may be reduced as a result of impaired social processing and emotional blunting (Duke and Begue 2015). For facial displays of emotion specifically, self-reported valences following alcohol consumption may mirror the inappropriate responses given by individuals scoring high in psychopathy (Wai and Tiliopoulos 2012).

## Method

### Participants

The present sample size was based on previous research (Duke and Begue 2015). Fifty participants comprising 33 females and 17 males (*M*_age_ *=* 21.60, *SD* = 4.65 years, age range 18–42 years, ethnicity: 89.58% Caucasian, 6.25% Mixed/multiple ethnic groups, 2.08% Asian British, 2.08% Black African) were recruited from the Plymouth University, School of Psychology student participant pool and participated for course credit. All participants were students enrolled on a psychology course at the university. All participants had normal or corrected-to-normal vision and the majority of participants were right-handed (84.1%). Two participants were excluded from the experiment; one having failed to see clearly in the non-moral virtual task as a result of vision problems and one having failed to complete the non-moral virtual task due to lack of understanding. As such, 48 participants comprising 31 females and 17 males (*M*_age_ *=* 21.44, *SD* = 4.63 years, age range 18–42 years) formed the final sample. All participants met inclusion criteria; they were not alcohol-naïve, not suffering from panic or anxiety attacks, not taking prescription medication that would be affected by alcohol consumption, they had no personal or family history of alcohol dependence, they did not report significant psychological problems, they were not pregnant or expecting to become pregnant, and they had not experienced aversive allergic reactions to alcohol consumption in the past. This research received ethical approval from the Plymouth University Ethics Committee.

### Experimental design

**Fig. 1:**
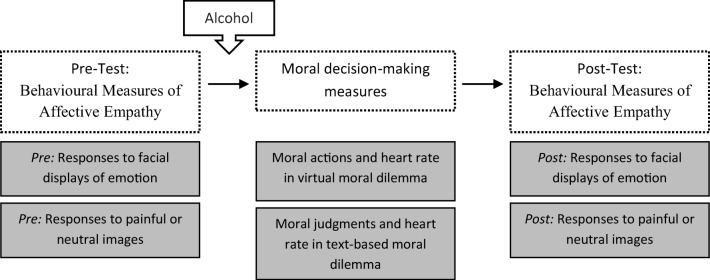
Primary outcome measures in experimental sequence. Alcohol is consumed (at varying dosages) after the first set of behavioural measures of affective empathy are completed. Note that each participant completes all of the tasks displayed (repeated measures variables)

Participants were randomly assigned to one of three conditions: placebo (*N =* 16, 10 females and 6 males (*M*_age_ *=* 22.13, *SD* = 6.43 years); low alcohol (*N =* 16, 10 females and 6 males (*M*_age_ *=* 20.06, *SD* = 2.82 years); high alcohol (*N =* 16, 11 females and 5 males (*M*_age_ *=* 22.13, *SD* = 3.91 years). A mixed-model design was adopted to examine whether there were differences between groups of individuals who consumed varying dosages of alcohol (placebo; low alcohol; high alcohol) across a set of repeated measures tasks (behavioural measures of affective empathy; moral decision-making measures). The main outcome measures were behavioural measures of affective empathy scores (self-reported valence to facial displays of emotion; self-reported intensity of pain pictured in images) before and after alcohol consumption, moral responses (moral actions simulated in a virtual moral dilemma; moral judgments in a text-based moral dilemma), and heart rate responses in both a virtual reality and text-based moral dilemma (see Fig. 1).

Secondary measures included personality trait assessments (to examine their relationship to responses in the behavioural measures of affective empathy prior to alcohol consumption) and manipulation checks and various control measures (of which full analyses can be found in the Supplementary Material).

### Personality measures

Prior to the experiment, participants were asked to fill out an electronic questionnaire comprising four self-report measures:

- The Levenson Self-Report Psychopathy Scale (LSRP) (Levenson et al. 1995) is a self-report measure of psychopathy intended for research purposes. It has a two-factor structure assessing both primary psychopathic traits (16 items, e.g. selfishness) and secondary psychopathic traits (10 items, e.g. impulsivity) in non-institutionalised populations (*α*s = .72–.84). The scale contains 26 items total, rated on a 4-point Likert scale (from 1 = *strongly disagree* to 4 = *strongly agree*). The scale includes items such as ‘For me, what’s right is whatever I can get away with’.
- The Hexaco-60 (Ashton and Lee 2009) is a personality inventory designed to assess six dimensions of personality. The inventory assesses the characteristics of Honesty-Humility (items 10), Emotionality (items 10), Extraversion (items 10), Agreeableness (items 10), Conscientiousness (items 10), and Openness to experience (items 10) (*α*s = .79–.82).^5^ The inventory contains 60 items with responses given on a 5-point Likert scale (from 1 = *strongly disagree* to 5 = *strongly agree*). The inventory contains items such as ‘I wouldn’t pretend to like someone just to get that person to do favours for me’.
- The Interpersonal Reactivity Index (IRI) (Davis 1983) is an inventory designed to measure dispositional empathy. It contains four subscales to measure Perspective Taking, Empathic Concern, Personal Distress, and Fantasy (*α*s = .72–.84). Perspective Taking is thought to relate to cognitive empathy whilst Empathic Concern is thought to relate to affective empathy. Personal Distress is often seen as a distinct conceptualisation to empathy (e.g. Batson 2009; Decety and Moriguchi 2007) and is included as a measure of ‘...self-oriented, egoistic’ reactions (Decety and Moriguchi 2007, p.17). The Fantasy subscale measures tendencies to relate to the feelings of fictitious characters. The inventory contains 28 items with responses given on a 5-point Likert scale (from A = *Does not describe me well* to E = *Describes me very well*). The scale contains items such as ‘I am often quite touched by things that I see happen’.
- The Self-Importance of Moral Identity Scale (Aquino and Reed 2002) provides a measure of moral identity. It contains two subscales that assess symbolisation (i.e. public dimension of moral identity) (5 items; *α* = .69) and internalisation (i.e. private dimension of moral identity) (5 items; *α* = .87). The inventory contains 10 items with responses given on a 5-point Likert scale (from A = *Does not describe me well* to E = *Describes me very well*). The scale contains items such as ‘It would make me feel good to be a person who has these characteristics’.

### Behavioural measures of affective empathy

In the present experiment, additional behavioural measures of affective empathy were included. These were completed by all participants pre- and post-intervention in a counterbalanced order:

#### Facial task

**Fig. 2:**
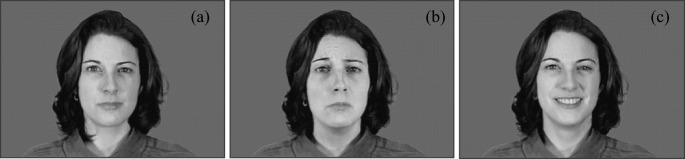
Example of neutral (**a**), sad (**b**), and happy (**c**) expression stimuli (Beaupré et al. 2000)

In an attempt to assess affective empathy, the Self-Assessment Manikin (SAM) (Bradley and Lang 1994) assesses an individual’s response to emotional stimuli rather than relying on self-report questionnaires. Adopting a procedure similar to that used in previous research (Wai and Tiliopoulos 2012), images depicting specific facial expressions (happy, sad, and neutral) were presented to participants (see Fig. 2). These images were sampled from the Montreal Set of Facial Displays of Emotion (MSFDE) (Beaupré et al. 2000) and comprised eight images per emotion, gender-balanced, and comprising only Caucasian faces. All images were presented in the same size and in greyscale. Following presentation, participants were asked to indicate how they felt towards the face on the SAM valence scale (1 (negative)–9 (positive)). In the pre-intervention task, four images of each emotion were presented to participants with the remaining 50% of images presented during the post-intervention task to prevent carryover effects.

#### Pain task

**Fig. 3:**
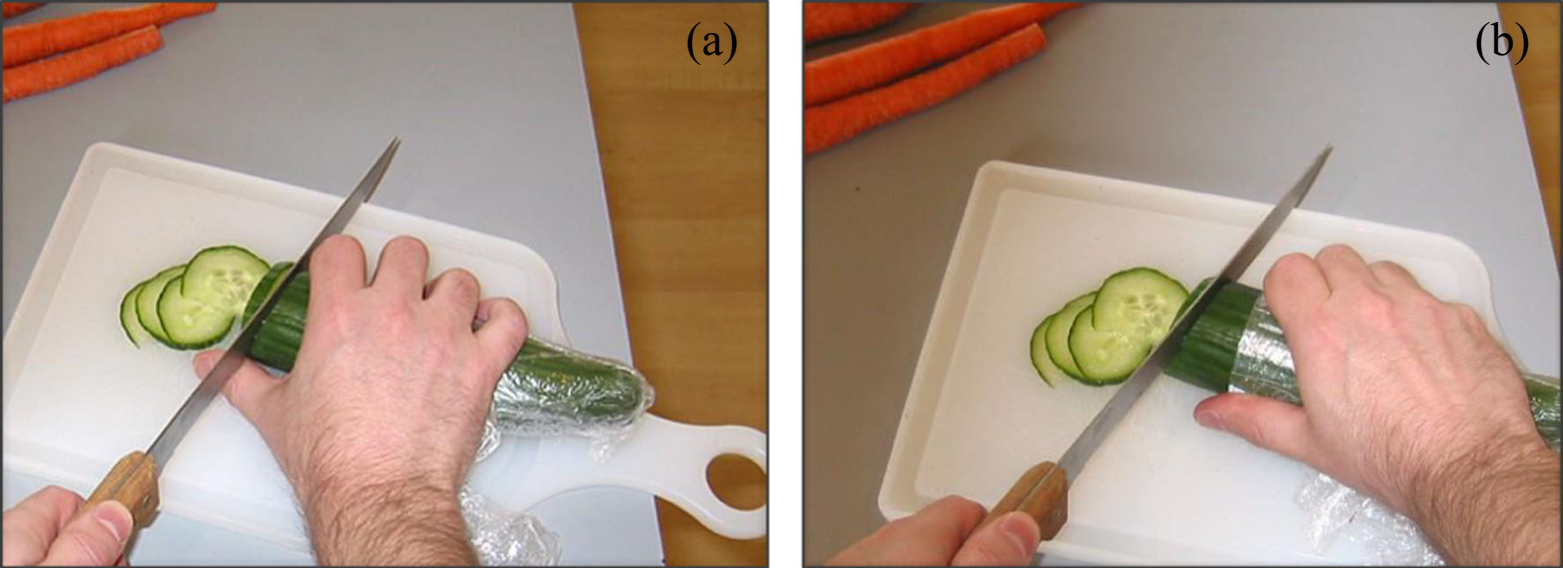
Example of painful stimuli (**a**) and non-painful stimuli (**b**) (Jackson et al. 2005)

The e*mpathy for pain* paradigm included in the present experiment has been adopted in previous research as a means of assessing affective responses when perceiving the pain of others (Jackson et al. 2005) and has been argued to be a way of investigating the processes involved in empathy (Decety and Jackson 2004). Adopting procedures from previous research (e.g. Jackson et al. 2005), images of hands and feet in painful and neutral conditions were presented to individuals (see Fig. 3). Following this, participants were asked to indicate on a visual analogue scale (VAS) (0 (no pain)–10 (worse pain ever)) the intensity of pain they thought the person in the image would feel in that situation. Our image group, sampled from an existing set (Jackson et al. 2005), comprised 18 painful images of familiar events and 18 neutral counterparts of the same events taken at ‘… angles that promoted first-person perspective’ (Jackson et al. 2005, p. 772). The types of pain included in these images were mechanical, thermal, and pressure-related with individuals in the images varying in both gender and age. All images were displayed in the same size and in colour. In the pre-intervention task, nine of the neutral and nine of the painful images were presented to participants with the remaining images presented during the post-intervention task to prevent carryover effects.

The presentation of facial displays of emotion and pain image blocks was counterbalanced across participants in both the pre-intervention and post-intervention completion of the tasks. No image was displayed more than once throughout the whole experiment.

### Moral decision-making measures

All participants completed a non-moral and moral action task and a non-moral and moral judgment task, both taken from Francis et al. (2016). For the action tasks, participants were presented with virtual reality scenarios using a head-mounted display (Oculus Rift 2). In the non-moral and moral virtual tasks, participants were given verbal instructions informing them that they would have the opportunity to engage with a virtual object using a joystick. Participants first completed the non-moral virtual task which involved pushing a virtual object (3D shape) upon hearing a tone. This task allowed a baseline measure of heart rate change to be collected (see physiological measures). It also allowed us to determine if changes in arousal in the subsequent virtual moral task were attributable to modality or moral content (Francis et al. 2016). For the moral action task, participants were presented with an audio-visual virtual reconstruction of the footbridge dilemma. The dilemma played out in real-time as described in Foot (1978) with the participant standing on a footbridge behind a large person. The scene was displayed in first person view. In the scenario, a modern train railcar approached from behind the participant, towards five virtual human agents standing on the tracks in front of the participant. Participants would have to decide whether to push the large person off the footbridge to stop the railcar’s progress or to allow the railcar to continue and kill the people standing on the track ahead. The following audio descriptions were played to participants within the virtual environment:

1. 30s*:* ‘Look behind you, a train is coming’.
2. 55s: ‘Hey I am too far away but if you want to save the people you could push the large person on to the tracks and derail the train. If you’re going to push them, do it now, but it is your choice’.

As in previous studies (see Francis et al. 2016; Francis et al. 2017a), participants were given 10 s to respond in the dilemma (by choosing to push with the joystick or by choosing to do nothing).

For the judgment task, participants were given a non-moral sample vignette to read which contained instructions displayed in the format of the pending dilemmas. Given that participants were completing both the moral action and moral judgment task, a validated^6^ and comparable moral dilemma to the *footbridge* dilemma was used in moral judgment task (see Francis et al. 2017a). This comparable moral dilemma was matched to the *footbridge* across the moral principles of benefit-recipient, inevitability, moral magnitude, physical contact, and personal force. This dilemma was embedded in nine distracter dilemmas; five were classified as personal and four as impersonal and were selected from those originally used in Greene et al. (2001). All dilemmas were presented electronically in a random order. As in previous studies (Francis et al. 2016; Francis et al. 2017b; Francis et al. 2017a), after each dilemma, participants were asked a morality question (‘Is it morally acceptable to [specific to the scenario]?’). After a response was given, a second behavioural question was displayed asking (‘Would you do it?’).^7^ Participants responded by selecting ‘Yes’ (Y- key) or ‘No’ (N- key) and these were then coded as utilitarian (yes) or non-utilitarian (no).

### Physiological measures

#### Alcohol

In order to assess and monitor the effects of alcohol in the low and high alcohol conditions, estimated blood alcohol levels (% BAC) were taken at specific intervals during the experiment from each participant’s breath air, using a portable breathalyser device (AlcoSense Pro Breathalyser and Alcohol Tester) utilised by UK police forces. The breathalyser measures the concentration of alcohol vapour in a single breath.

#### Moral decision-making tasks

Heart rate was recorded using the equipment and procedure adopted in Francis et al. (2016). A Cateye-PL-6000 heart rate monitor was attached to participants via an ear clip. As outlined in previous research, heart rate change (bpm) can be both abrupt and gradual (Francis et al. 2016) and so heart rate readings were taken at the onset and offset of all non-moral and moral tasks. The duration between onset and offset of tasks was dependent on the task type (non-moral; moral) and was determined by reading speed in the judgment tasks (see Francis et al. 2016 for a full description of this sampling procedure). Heart rate sampling was completed with the primary aims of assessing whether arousal was modality or moral specific.^8^

### Procedure

#### Pre-intervention

**Fig. 4:**
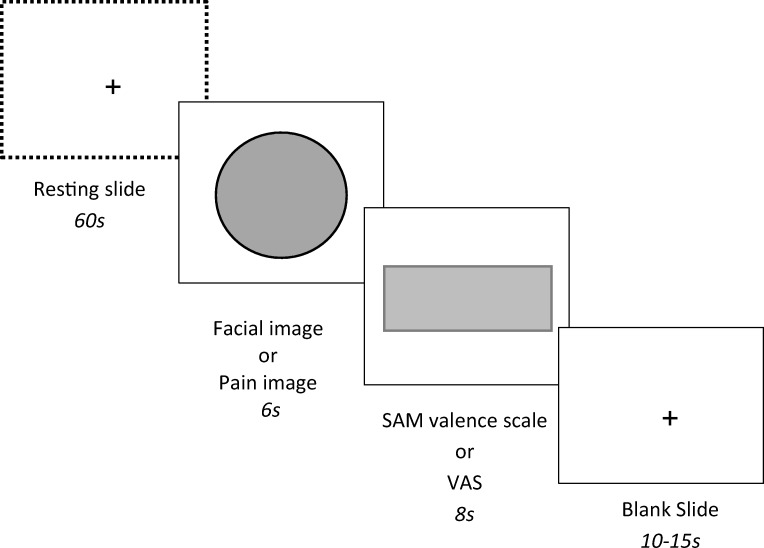
Experimental procedure in behavioural measures of affective empathy (facial and pain tasks). The same procedure was adopted in both the pre-intervention and post-intervention behavioural measures of affective empathy

Prior to arriving at the experiment, participants were reminded to refrain from drinking alcoholic beverages within 12 h of the experiment beginning. All conditions first completed the personality trait assessments, a pre-questionnaire assessing their gaming experience (weekly hours of video game playing and number of times playing games annually) and a subjective mood visual analogue scale (100 mm long) assessing disinhibition and positive affect (Duke and Begue 2015). Participants then completed the behavioural measures of affective empathy. Participants were seated 50 cm away from a PC. At the beginning of the task, a resting slide appeared on-screen and participants were instructed to look at the fixation cross at the centre of the screen. Sixty seconds from the onset of the resting slide, the first stimulus appeared. Following an existing procedure (Partala and Surakka 2003), each image stimulus was presented for 6 s. After image offset, the relevant scale was presented for 8 s to be completed by participants (SAM valence scale for facial displays of emotion or VAS for pain images). Participants used the computer mouse with their right hand to select a rating along the given scale. Following scale offset, a blank slide with a fixation cross would be displayed for a randomised interval of 10–15 s before the next image stimulus was delivered to prevent anticipation of stimuli (see Fig. 4)**.** Following completion of the pre-intervention behavioural measures of affective empathy, participants were given an additional questionnaire to complete which assessed their alcohol consumption (units per week) and their current weight (kg).^9^ Participants were then randomly allocated to one of the three conditions: placebo, low alcohol, or high alcohol. An estimate baseline BAC was then taken (participants were asked to blow into a sterile tube attached to the portable breathalysing device).

#### Intervention

In an attempt to reach target BAC levels predefined in existing research and subsequently shown to affect moral decision-making: 0.038–0.04% (Duke and Begue 2015), the high alcohol condition received 0.80 g/kg vodka (37.5% alcohol by volume). The low alcohol condition received a dose of 0.40 g/kg vodka (37.5% alcohol by volume) to produce lower BAC levels, allowing a systematic investigation of the effect (if present) across a range of BAC levels. All alcoholic drinks were mixed with two-part lemonade and were flavoured with fresh lime juice. Participants in the placebo condition were given lemonade flavoured with lime and alcohol was sprayed around the edge of the glass in order to provide an alcohol odour, ensuring that condition assignment was unknown. Alcohol expectancy has been shown to influence social behaviours (e.g. Assefi and Garry 2003) by providing an excuse for individuals to engage in inhibited social behaviours and the ability to justify these behaviours. Given these expectancy effects, additional alcohol cues such as floating a small amount of alcohol on top of each placebo glass (e.g. Roberts et al. 2012) were not adopted here to avoid participant expectation confounding task performance. The aim of this manipulation was to ensure that condition assignment was unknown and not to deceive placebo participants into accepting that they had consumed alcohol. In all conditions, participants were given 10 min to consume the beverage. In order to control for awareness of condition assignment, an awareness check was performed asking participants whether they knew which condition they had been assignment to and if so, how they knew. A waiting period of 20 min followed in order for alcohol to be absorbed into the blood and to reach a predefined optimal level.^10^ Following this, a second BAC reading was taken (estimated peak BAC).

#### Post-intervention

All participants completed a second subjective mood assessment of disinhibition and positive affect, followed by the moral action and judgment tasks. The order of the moral tasks was counterbalanced. Participants then completed the post-intervention behavioural measures of affective empathy using a procedure identical to that of the pre-intervention behavioural measures of affective empathy. Following completion of this task, a final estimate BAC reading was taken. Participants were invited to leave the experiment after their BAC level had returned to a predetermined limit (< 0.01%).

## Statistical analysis

Given the mixed model experimental design adopted here, primary outcome measures were analysed as follows:

### Moral responses and alcohol

In order to compare moral judgments and moral actions, simulated moral actions in the virtual version of the footbridge dilemma were compared with the moral judgments made in response to the text-based counterpart. Given that responses to the moral judgment task and moral action task were binary (yes/utilitarian; no/non-utilitarian), generalised estimating equations (GEE) were performed using a binary logistic model with task (judgment task; action task) as within-subjects factor and condition (placebo; low alcohol; high alcohol) as between-subjects factor. Two analyses were carried out, the first using the morality question in the judgment task and the second using the behavioural question in the judgment task. This analysis was designed to compare moral actions in virtual reality and moral judgments in text-based vignettes; hence, the morality and behavioural questions were referenced in separate analyses (as both are self-reported judgment deriving from the same text-based moral dilemma). Generalized estimating equations (GEE) are an extension of generalized linear models (GZLM). Like GZLM, it allows analysis of scale, count, or binary responses (residuals can follow a non-normal distribution) but also allows analysis of repeated measures variables (or more broadly, correlated observations) which GZLM does not. Given that GEE enable analysis of binary responses and repeated measures variables, it is used here to analyse binary moral responses (utilitarian; non-utilitarian) across the repeated measures moral decision-making tasks (virtual moral dilemma; text-based moral dilemma) in the three alcohol groups (placebo; low alcohol; high alcohol). This analysis has been used in previous research comparing moral action and judgment in the same individuals (Francis et al. 2017a). Given that these analyses are dealing with binary data, Cohen’s *h* is included as a measure of effect size as it concerns the difference between two proportions and the same rules of thumb for interpreting the size of Cohen’s *d* apply to Cohen’s *h*.

### Heart rate and alcohol

In all groups, changes in heart rate were calculated by subtracting the heart rate readings (bpm) taken at the offset of the moral (and non-moral) tasks from those taken at the onset of the moral (and non-moral task) tasks. Heart rate changes were analysed using a mixed model ANOVA although the ratio of the greatest and least variance in heart rate change was > 3 and as such, this analysis was repeated, and findings replicated using generalised estimating equations, which does not assume homogeneity of variance (see Supplementary Material).

Note that additional analyses were performed for these primary outcome measures to examine the relationship between individual differences in alcohol absorption and moral responses and individual differences in alcohol absorption and heart rate changes.

### Alcohol and behavioural measures of affective empathy

#### Facial task

The SAM was used to assess self-reported valence to facial emotions and subsequent affective empathy (Wai and Tiliopoulos 2012). Valence scores were calculated by averaging self-reported valence scores (1 (*negative*)–9 (*positive*)) across each emotion set of facial expressions (neutral; happy; sad) for the pre-intervention and post-intervention tests (see Supplementary Material for descriptive statistics).

#### Pain task

The pain task was used to assess affective empathy for pain (Jackson et al. 2005). Empathy for pain scores were calculated by averaging the responses given on the VAS (0 (*no pain*)–10 (*worse pain ever*)) for neutral and painful images for the pre-intervention and post-intervention tests (see Supplementary Material for descriptive statistics).

In order to examine the effects of the alcohol intervention on these behavioural measures of affective empathy, separate three-way mixed ANOVAs were performed for the facial task and pain task. If behavioural measures of affective empathy (valence towards faces or pain responses) were affected by alcohol consumption, secondary analyses were performed to determine whether these changes affected moral decision-making. For the facial task, changes in valence were calculated by taking the difference between mean valence scores for each facial expression between the pre- and post-intervention tests. Prior to calculating change scores and to account for baseline differences, a one-way ANOVA found no differences between valence scores for each facial expression between conditions in the pre-intervention test (*p*s > .289). For the pain task, changes in mean empathy for pain scores were calculated in the same way by taking the difference between VAS scores for both image types (neutral; painful) between the pre- and post-intervention. There were no differences between VAS scores for both image types between conditions in the pre-intervention test (*p*s > .642). The relationship between these change scores and moral actions and moral judgments was subsequently analysed using point-biserial correlations. In a final analysis of these behavioural measures of affective empathy and in order to determine if these behavioural assessments of affective empathy were related to self-report measures of affective empathy, psychopathy, and associated traits, bivariate correlations were performed between traits and self-reported valence to facial emotions collected in the pre-intervention test and between traits and self-reported responses to neutral and painful images collected in the pre-intervention test, prior to the alcohol intervention. This was done to validate the behavioural measures of affective empathy included here as in previous research (Wai and Tiliopoulos 2012). The post-intervention behavioural measures of affective empathy were not included in this analysis given the potential mediating effects of alcohol consumption on online performances in these tasks.

## Results

### Control variables and checks

BAC levels were significantly different between all conditions, (*F*(2, 45) = 51.97, *p* < .001, *η*_*p*_^*2*^ = .70), with average peak BAC levels (%) highest in the high alcohol condition (*M* = 0.03%, *SD* = 0.01, Range = 0.01–0.05%), moderate in the low alcohol condition (*M* = 0.01%, *SD* = 0.01, Range = 0–0.03%), and as expected, absent in the placebo condition. Reported awareness of condition assignment was not associated with either moral actions or moral judgments (*p*s > .388). Across conditions, there were no differences in self-reported drinking habits (*p* = .328) and drinking habits did not correlate with peak BAC level (*p* = .975). With previous research suggesting that the relationship between alcohol and moral decision-making may be influenced by feelings of disinhibition or positive affect (Duke and Begue 2015), subjective mood ratings (disinhibition; positive affect) were compared before and after the alcohol intervention. Analysis revealed that self-reported disinhibition was not different between conditions (*p* = .740) or following the intervention (*p* = .938). Positive affect was significantly lower after the intervention, (*F*(1, 45) = 9.18, *p* = .004, *d* = 0.32) but was not associated with either moral actions (*p* = .673) or moral judgments (*p*s > .175). Following these checks, awareness checks, drinking habits, and subjective mood were not included in further analyses (for full details regarding analyses of control variables and checks, see Supplementary Material).

### Moral responses

Across alcohol and placebo conditions, the proportion of utilitarian responses was higher when simulated action was required in virtual reality compared with when judgment was required in the text-based counterpart (see Table 1).

**Table 1:** Percentage of utilitarian responses in moral judgment and action tasks

	Moral judgment task		Moral action task
Condition	Morality question	Behavioural question	
Placebo	25%	25%	75%
Low alcohol	18.75%	6.25%	68.75%
High alcohol	6.25%	6.25%	68.75%

**Fig. 5:**
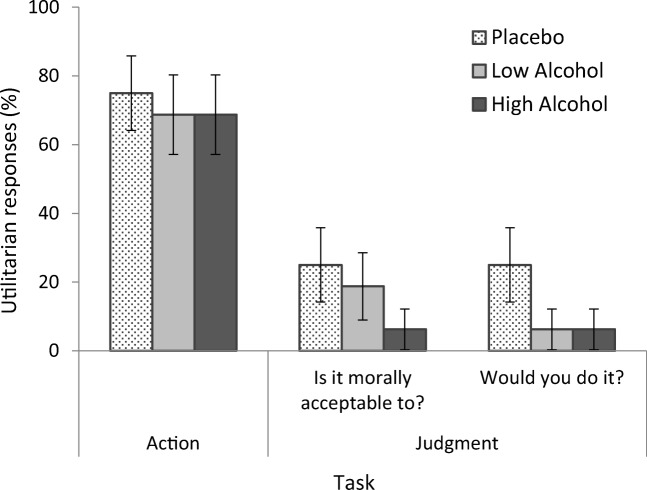
Utilitarian responses (%) in the moral action task (virtual footbridge) and the moral judgment task (text-based footbridge counterpart) in the placebo, low alcohol, and high alcohol conditions. In the judgment task, participants were asked whether the action was morally acceptable and whether they would do it. A greater number of utilitarian outcomes were endorsed in the moral action task (Although GEE analyses revealed no significant response differences between conditions, the differences between conditions in their responses to the judgment task appeared to be large (e.g. 6.25% versus 25% utilitarian responses). As such, we carried out additional separate chi-square tests comparing responses to the morality question and the behavioural question between conditions. These supported GEE analyses showing no significant differences between conditions in their moral judgments made in response to either the morality question (*p* = .492) or behavioural question (*p* = .333)). Error bars represent ± 1 SE_p_.

GEE analyses were performed using a binary logistic model with task (judgment task; action task) as within-subjects factor and condition (placebo; low alcohol; high alcohol) as between-subjects factor (see Fig. 5). When referencing the morality question, analysis revealed a main effect of task, (Wald *X*^2^[1] = 27.18, *p* < .001, *h* = − 1.15), with a greater proportion of utilitarian responses overall in action tasks compared with judgment tasks. There was no main effect of condition (*p* = .470) and no interaction (*p* = .566). When referencing the behavioural question, analysis revealed a main effect of task, (Wald *X*^2^[1] = 24.90, *p* < .001, *h* = − 1.27), with a greater proportion of utilitarian responses overall in action tasks compared with judgment tasks. There was no main effect of condition (*p* = .286) and no interaction (*p* = .480).^11^

Following statistical analyses adopted in previous research (Duke and Begue 2015), analyses were also carried out using BAC level as a predictor of moral responses (non-utilitarian; utilitarian) in both the moral action task and moral judgment task. Peak BAC level was not a significant predictor of moral responses in the virtual moral action task (*p* = .575) or the moral judgment task when referencing both the morality and behavioural question (*p*s > .109).

#### Heart rate responses

Mean heart rate change was highest for the moral action task (virtual *footbridge* dilemma) across conditions. Heart rate change decreased for the moral judgment task (text counterpart dilemma) and both the action and judgment non-moral tasks.

**Fig. 6:**
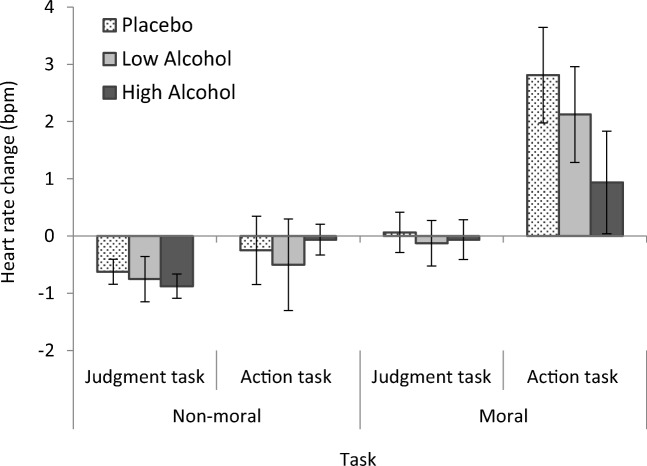
Mean heart rate change (bpm) for non-moral and moral task type in judgment and action tasks by condition. Increased heart rate changes were observed in the virtual moral action task across conditions**.** Error bars represent ± 1 SE

A mixed ANOVA was conducted on heart rate changes with task (judgment task; action task) and type (non-moral task; moral task) as within-subjects factors and condition (placebo; low alcohol; high alcohol) as the between-subjects factor. Analysis revealed a main effect of task, (*F*(1, 45) = 23.12, *p* < .001), a main effect of type, (*F*(1, 45) = 20.70, *p* < .001), and a significant interaction of type *×* task, (*F*(1, 45) = 5.92, *p* = .019) (see Fig. 6). There was no main effect of condition (*p* = .436) and no further interactions (*p*s > .320).

To further investigate the interaction of type *×* task, simple effects analyses were performed comparing heart rate changes in non-moral and moral tasks within both judgment and action tasks. A significant difference was found between non-moral and moral tasks in the judgment task across groups, (*F*(1, 45) = 8.11, *p* = .007, *d* = − 0.63) and in the action task across groups, (*F*(1, 45) = 14.53, *p* < .001, *d* = − 0.80) with greater heart rate changes observed in moral tasks. There was a significant difference in heart rate change between the judgment and action task but for the moral tasks only, (*F*(1, 45) = 17.21, *p* < .001, *d* = − 0.76) with greater heart rate changes observed overall in the virtual moral action task. Heart rate change for the non-moral tasks was not significantly different between action and judgment tasks (*p* = .129).

**Fig. 7:**
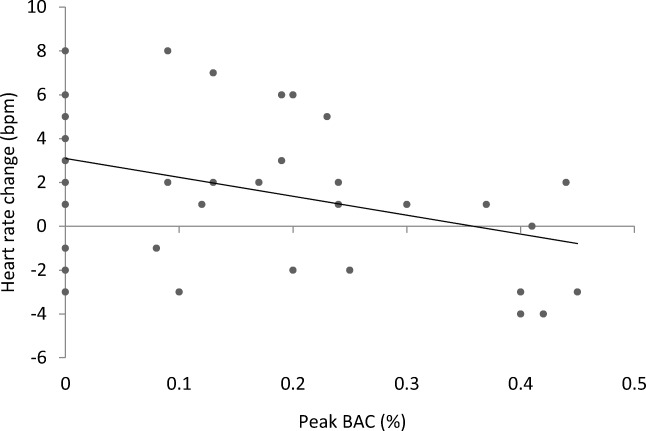
Heart rate change (bpm) in the moral action task (virtual footbridge) plotted against peak BAC levels (%). Peak BAC was a negative predictor of heart rate change in the virtual moral action task. Linear regression trend line, *R*^2^ = .14

In further analyses accounting for variation in alcohol absorption, bivariate correlations were carried out to determine whether heart rate change in tasks was associated with peak BAC levels. BAC levels were not correlated with heart rate change in the moral judgment task (*p* = .789) or the judgment and action non-moral tasks (*p*s > .536). Peak BAC level had a moderate negative correlation with heart rate change in the moral action task, (*r*(46) = − .37, *p* = .009) (see Fig. 7) and when entered into a univariate linear regression, was found to explain 13.8% of the variance in the model, (*R*^2^ = .138, *F*(1, 46) = 7.36, *p* = .009) when predicting this heart rate change (*β* = − .37, *p* = .009).

### Behavioural measures of affective empathy

#### Valence and alcohol

A three-way mixed ANOVA was performed to determine the effects of condition (placebo; low alcohol; high alcohol), emotion (neutral; happy; sad), and test (pre-intervention; post-intervention) on self-reported valence to faces. Analysis revealed a main effect of emotion, (*F*(2, 90) = 141.04, *p* < .001), a significant two-way interaction of test *×* emotion, (*F*(2, 90) = 5.95, *p* = .004), and a statistically significant three-way interaction between condition *×* test *×* emotion, (*F*(4, 90) = 6.03, *p* < .001). There was no main effect of test (*p* = .195) or condition (*p* = .990) and no further interactions (*p*s > .463).

**Fig. 8:**
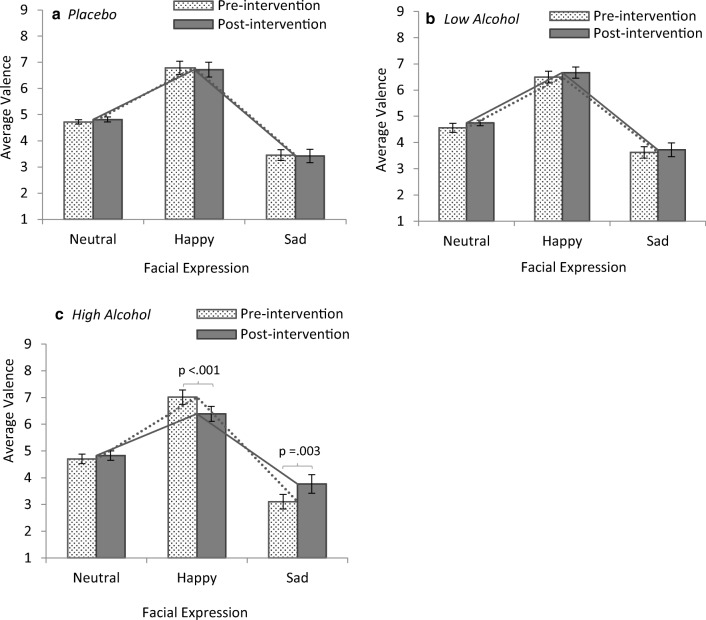
Simple interaction effects showing average self-reported valence to facial expressions (1 (*negative*)–9 (*positive*)) in the (*a*) placebo condition, (*b*) low alcohol condition, and (*c*) high alcohol condition. A significant interaction effect was found for the high alcohol condition and for happy and sad facial expressions only. This interaction can be seen in the different slopes of the pre-intervention (dotted line) and post-intervention (solid line) valence in the (*c*) high alcohol condition. Error bars represent ± 1 SE

In order to examine the higher-order interaction further, simple effects tests follow. Statistical significance of simple two-way interactions and follow-up simple main effects were accepted at a Bonferroni-adjusted level (*p* = .017). Analysis revealed a statistically significant two-way interaction of test *×* emotion in the high alcohol condition, (*F*(2, 30) = 21.21, *p* < .001) (see Fig. 8c) but not in the placebo (*p* = .665) (see Fig. 8a) or low alcohol conditions (*p* = .933) (see Fig. 8b). In order to investigate this interaction further, the effect of test (pre-intervention; post-intervention) was examined for each emotion (neutral; happy; sad) using simple effects tests. There was a statistically significant simple main effect of test for the high alcohol condition in reported valence to happy faces and sad faces but not in valence towards neutral faces (*p* = .309). For participants in the high alcohol condition, valence towards happy faces was significantly lower (more negative) in the post-test after alcohol consumption, (*t*(15) = 5.18, *p* < .001, *d* = 1.29) and valence towards sad faces was significantly higher (more positive) in the post-test after alcohol consumption, (*t*(15) = − 3.46, *p* = .003, *d* = − 0.87) (see Fig. 8c).

#### Empathy for pain and alcohol

A three-way mixed ANOVA was performed to determine the effects of condition (placebo; low alcohol; high alcohol), image type (neutral; painful), and test (pre-intervention; post-intervention) on VAS scores assessing empathy for pain. Analysis revealed a significant main effect of image type, (*F*(1, 45) = 1221.51, *p* < .001), and a significant two-way interaction of test *×* image type, (*F*(1, 45) = 9.23, *p* = .004). There was no main effect of test (*p* = .056), no main effect of condition (*p* = .835), and no further interactions (*p*s > .500).

**Fig. 9:**
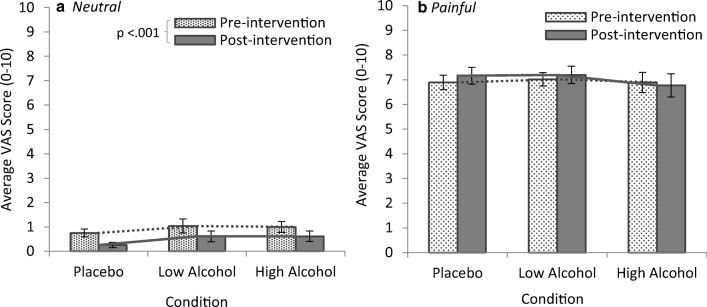
Bar graphs showing average empathy for pain scores on the VAS (0 (*no pain*)–10 (*worse pain ever*) in response to (*a*) neutral images and (*b*) painful images. A significant interaction effect revealed higher VAS scores for painful images across pre- and post-intervention tests and significantly lower VAS scores for neutral images in the post-test. This interaction can be seen in the different slopes of the VAS scores for painful images versus neutral images and in the different slopes of the pre-intervention (dotted line) and post-intervention (solid line) VAS scores in the (*a*) neutral images. Error bars represent ± 1 SE

To further investigate the interaction between test *×* image type, simple effects analyses were performed comparing empathy for pain scores for neutral and painful images within both the pre-intervention and post-intervention tests. A significant difference was found between pain scores for neutral and painful images in both the pre-test (*F*(1, 45) = 1031.75, *p* < .001, *d* = − 5.35) and post-test (*F*(1, 45) = 943.13, *p* < .001, *d* = − 5.38) with painful images eliciting higher VAS scores overall. In the post-test, empathy for pain scores in response to neutral images was significantly lower compared with the pre-test, (*F*(1, 45) = 14.52, *p* < .001, *d* = 0.51) (see Fig. 9).

#### Affective empathy and moral responses

Following the finding that valence towards sad and happy faces was affected by alcohol consumption, a point-biserial correlational analysis was performed revealing that changes in self-reported valence towards happy and sad faces between the pre- and post-intervention tests were not associated with moral actions (*p*s > .651) or moral judgments (*p*s > .372). Given the test × emotion × condition interaction, partial correlations controlling for BAC levels were also performed revealing no relationship between changes in self-reported valence towards happy and sad faces and moral responses (*p*s > .132).

Changes in empathy for pain scores between the pre- and post-interventions for neutral and painful images were not associated with either moral actions (*p*s > .114) or moral judgments (*p*s > .344).

#### Affective empathy and traits

Moderate correlations showed that individuals scoring higher in primary psychopathy felt more positively when looking at sad facial expressions, (*r*(46) = .39, *p* = .007) and more negatively when looking at happy facial expressions, (*r*(46) = − .34, *p* = .017). Honesty-Humility, on the other hand, correlated negatively with valence towards sad facial expressions, (*r*(46) = − .47, *p* = .001) and positively with valence towards happy facial expressions, (*r*(46) = .35, *p* = .015). Additionally, individuals scoring higher in Internalisation felt more positively towards happy facial expressions, (*r*(46) = .29, *p* = .048) and individuals with higher Empathic Concern scores reported feeling more negative towards sad facial expressions, (*r*(46) = − .30, *p* = .037) (see Supplementary Material for table of trait correlations).

Moderate correlations revealed that individuals scoring higher in primary psychopathy had lower empathy for pain scores when looking at painful images, (*r*(46) = − .35, *p* = .016). Empathic Concern, on the other hand, correlated positively with empathy for pain scores on the VAS in response to painful images, (*r*(46) = .31, *p* = .03). Individuals scoring higher in Honesty-Humility reported greater empathy for pain scores when looking at neutral images, (*r*(46) = .32, *p* = .025) (see Supplementary Material for table of trait correlations).

## General discussion

Overall, greater utilitarian endorsements were observed when simulated action was required in virtual reality, compared with when moral judgments were required in a text-based counterpart dilemma. Whilst alcohol consumption altered self-reported performances in behavioural measures of affective empathy and heart rate responses in the virtual moral dilemma, in the present study, alcohol intake did not affect moral actions or moral judgments. These results contrast with previous findings investigating acute alcohol effects on moral decision-making.

### Moral actions versus judgments

Participants in the present experiment demonstrated moral inconsistency; greater utilitarian actions were observed in the virtual *footbridge* dilemma, with fewer utilitarian judgments observed in the text-based counterpart, regardless of condition assignment. These results corroborate existing virtual research that demonstrates disparity between saying and doing (e.g. Francis et al. 2016; Francis et al. 2017b; Francis et al. 2017a; McDonald et al. 2017; Patil et al. 2014). In previous virtual research, the preference for simulating characteristically utilitarian actions in virtual reality has been interpreted through two accounts. Frame of reference accounts theorise that egocentric perspectives and subsequent self-interested motives drive moral actions as individuals consider the self-relevant consequences of their own actions (Francis et al. 2017b; Tassy et al. 2013a; Tassy et al. 2013b). Judgments, on the other hand, are theorised to rely on allocentric evaluations and subsequently, cultural norms (Tassy et al. 2013a; Tassy et al. 2013b). Contextual saliency accounts argue that the physical features in virtual scenarios allow individuals to ‘see’ potential victims in a moral dilemma, and this then results in greater negative emphasis being placed on witnessing victims die than on performing harmful actions (Francis et al. 2016; Francis et al. 2017b; Patil et al. 2014).

With regard to the alcohol intervention in the present experiment, we had hypothesised alternative outcomes based on divergent streams of research seeking to understand the roles of deliberation versus social processing in moral decision-making (e.g. Duke and Begue 2015; Greene et al. 2001; Patil 2015). Based on a deliberation-focused hypothesis, with alcohol increasing emotional reactivity and decreasing cognitive functioning, non-utilitarian preferences would be predicted (Greene et al. 2001). Alternatively, previous research has argued that alcohol intake results in deficits in social processing but more specifically, reduced aversion to harm, subsequently resulting in an increase in utilitarian moral judgments (Duke and Begue 2015).

In terms of moral actions, we outlined similar divergent hypotheses. If dual process theories of moral judgment (Greene et al. 2001) transfer to the domain of moral action (Navarrete et al. 2012), the deliberation hypothesis might also transfer to moral actions, resulting in fewer utilitarian endorsements. However, given evidence that moral action and judgment are partially distinct (e.g. Tassy et al. 2013b) and that moral inconsistency is often present in populations with social deficits (e.g. Cima et al. 2010; Patil 2015), there could be a preference for greater utilitarian actions after alcohol consumption following the social processing hypothesis. However, in the present experiment, neither moral judgments nor moral actions were affected by alcohol consumption. As such, these results cannot provide support for either the social processing hypothesis (Duke and Begue 2015) or the deliberation-based hypothesis (Greene et al. 2001).

### Alcohol and arousal

Supporting previous findings, heart rate changes were highest for virtual reality moral tasks across conditions (e.g. Francis et al. 2016). When taking BAC levels into account, increased BAC levels were associated with reduced arousal responses in the virtual reality moral task only. This supports the theory that virtual reality paradigms can prompt realistic physiological responses (e.g. Parsons 2015) and the theory that alcohol may trigger affective processing deficits in emotionally aversive situations (Duke and Begue 2015).

### Empathy and psychopathy

Pre-intervention behavioural affective empathy tasks were validated against existing personality trait assessments. Replicating previous research (Ali et al. 2009; Wai and Tiliopoulos 2012), primary psychopathy was negatively associated with self-reported valence towards positive faces and positively related to self-reported valence towards negative faces. Wai and Tiliopoulos (2012) argue that the presentation of facial displays of emotion using the SAM (Bradley and Lang 1994) may provide a more accurate measure of affective empathy than trait questionnaires. They argue that picturing another’s emotions generates an emotional contagion. Subsequently, the way in which an individual then feels (negatively or positively) about this display of emotion is an empathic measure derived from the appropriateness of that reaction (Wai and Tiliopoulos 2012). The finding that individuals scoring high in primary psychopathy demonstrate ‘… inappropriate responding’ to sad and happy faces (Wai and Tiliopoulos 2012, p. 797) reflects a deficit in this empathic contagion. The present experiment also extends these findings, having observed the opposite trend in individuals scoring high in traits negatively correlated with the Dark Triad, including Honesty-Humility, Empathic Concern, and Internalisation. In these cases, emotional responses to facial displays of emotion were appropriately aligned, with happy faces motivating self-reported positive valence and sad faces motivating negative valence.

We had also hypothesised that primary psychopathy would negatively correlate with empathy for pain scores. This was supported in the present findings; individuals scoring higher in trait primary psychopathy demonstrated less intensity when rating the pain of others. Extending from this, the inversely related trait, Empathic Concern, was positively correlated with empathy for pain. This aligns with previous research showing that individuals scoring higher in Empathic Concern demonstrate a high level of care and consideration for the welfare of others (e.g. Davis 1983). Unexpectedly, Honesty-Humility positively correlated with empathy for pain scores in neutral images. There are a few explanations for this association. Firstly, follow-up analysis revealed that empathy for pain scores between neutral and painful images was positively correlated in the pre-intervention task, (*r*(46) = .37, *p* = .009) and post-intervention task, (*r*(46) = .33, *p* = .022) suggesting that similar mechanisms drive ratings of pain or anticipated pain in neutral images. Secondly, higher Honesty-Humility has been associated with lower health- and safety-related risk-taking (Weller and Tikir 2011) which, in this instance, may have intensified the anticipation of harmful outcomes pictured in neutral images.

### Alcohol and empathy

Given that alcohol is thought to diminish aversion for harm and hinder social processing (e.g. Carmona-Perera et al. 2014; Duke and Begue 2015), we hypothesised that alcohol consumption would impair performance in the SAM as a result of these social impairments. This was supported in the present experiment; individuals receiving a high dosage of alcohol reported feeling more positively towards sad faces and more negatively towards happy faces in the post-intervention facial SAM task. These inappropriate responses reflect those of individuals scoring high in primary psychopathy (Wai and Tiliopoulos 2012) and support the theory that alcohol impairs components of affective empathy (Duke and Begue 2015). These results are in line with the finding that affective empathy is principally impaired in alcohol-dependent individuals (Maurage et al. 2011; Marinkovic et al. 2009) and addicted patients (e.g. Ferrari et al. 2014). However, in terms of moral responses, differences in the pre- and post-intervention facial behavioural measures of affective empathy did not relate to either moral actions or moral judgments when controlling for alcohol consumption. Despite supporting the association between alcohol and impairment of affective empathy (Duke and Begue 2015), this impairment did not result in utilitarian decision-making, as the social processing hypothesis would predict.

Further, given evidence that harm aversion plays a mediating role in personality traits associated with making supposedly utilitarian endorsements (Patil 2015), evidence for the role of these traits in moral inconsistency (Cima et al. 2010), and the theory that alcohol reduces harm aversion (Duke and Begue 2015), we had predicted that alcohol consumption would reduce affective empathy towards individuals in painful circumstances. However, alcohol dosage did not affect empathy for pain scores in the present experiment. This supports existing research findings that pain intensity ratings are not affected by acute alcohol consumption despite empathic neural activity for pain being reduced (Hu et al. 2017). Empathy for pain scores for neutral images was significantly lower following the intervention across all conditions regardless of alcohol intake but it is likely that this finding reflects a familiarity effect as individuals became aware of the distinction between painful and neutral images in the present investigation.

### Alcohol, empathy, and utilitarianism

It is important to raise an alternative interpretation, based on the present experiment’s findings, to the proposed association between affective empathy and utilitarian moral decision-making. Previous research has identified evidence of a relationship between anti-social traits, and simulated moral actions (Francis et al. 2016) and the power of these simulated actions (Francis et al. 2017b). We expanded these investigations using behavioural assessments of affective empathy given evidence that the relationship between psychopathy and utilitarian decision-making derives from empathic deficits (e.g. Glenn et al. 2009) and subsequent diminished aversion to performing harmful actions (e.g. Patil 2015).

However, in the present experiment, affective empathy was successfully manipulated in a facial responding task following alcohol consumption, but this did not affect moral decision-making. This may suggest that is not only the un-empathic facets of traits such as psychopathy that drive utilitarian moral decision-making but perhaps other facets. For example, psychopaths have been found to demonstrate low anxiety and fearlessness (e.g. Miller et al. 2008) which might instead explain their diminished aversion to harm and tendency to respond in a utilitarian manner. Indeed, Koenigs et al. 2012 found that low-anxious psychopaths (with inhibitory deficits) endorsed a greater proportion of utilitarian moral judgments in personal moral dilemmas when compared with high-anxious psychopaths and this relationship has also been evidenced in psychopharmacological investigations in which anti-anxiety drugs (Lorazepam) have reduced harm aversion and subsequently resulted in greater utilitarian endorsements (Perkins et al. 2013). The low anxious facets of primary psychopathy are theorised to reflect emotional and inhibitory deficits (Koenigs et al. 2012) that compromise conditionability of moral norms (Blair 1995) and subsequently reduce aversion to harm. For example, when facing a punishment following a transgression, we feel anxious, and this subsequently conditions us to avoid future transgressions. A diminished anxiety response is thought to compromise this conditioned response (e.g. Blair 1995). Again, evidence in this area has been mixed (e.g. Schmitt and Newman 1999; Visser et al. 2012) with research also highlighting the moderating role of aggression, rather than trait anxiety, in this relationship (Choe and Min 2011; Gao and Tang 2013). In the present investigation, we did not include measures of anxiety or aggression and so this theory remains speculative. However, with research suggesting a critical role for these facets in action aversion deficits, future research should consider incorporating these assessments in both moral judgment and moral action paradigms.

It should also be noted that the relationship between acute alcohol effects and utilitarian moral decision-making found in previous research (Duke and Begue 2015) may also derive from social awareness or social influence. Duke and Begue (2015) collected moral judgments made in response to the *footbridge* dilemma in bars. These social settings may have influenced moral judgments in a number of ways. Firstly, the disinhibited atmosphere may have made the perception of hypothetical trolley problems less serious, with previous research suggesting that these scenarios can be perceived as humorous (e.g. Bauman et al. 2014). Alternatively, social pressures may have resulted in individuals acting in a way that they felt was publicly acceptable under social expectation (e.g. Gold et al. 2015). As such, future research examining the acute effects of alcohol intake on moral decision-making might consider including control measures such as social desirability and self-awareness scales.

### Moral inconsistency

Whilst the within-subjects design of the present experiment allowed a direct comparison between moral judgments and moral actions made by the same individual (Patil et al. 2014), it might be argued that the comparison of moral judgments and moral actions is limited, given that each paradigm incorporated a different hypothetical moral dilemma. We chose not to include the footbridge dilemma in both the virtual- and text-based tasks to remove any potential carry-over effects (Bartels et al. 2015). Importantly, in previous validation studies (see Francis et al. 2017a), we did not find a significant difference between responses to the footbridge dilemma and the modified dilemma, suggesting that it could be utilised as a reliable comparable dilemma. Further, no order effects were found based on the presentation of moral judgment and action tasks, suggesting that utilising different dilemmas did prevent potential carry-over effects.

### Methodological considerations

It is also important to highlight limitations of the present methodology. Firstly, no measure of cognitive functioning or executive functioning was included in the present experiment, a criticism mirroring that of previous research (Duke and Begue 2015). This is significant given the mediating effect of executive functioning in the relationship between alcohol and aggression (e.g. Giancola 2000, 2004; Godlaski and Giancola 2009) and alcohol-related aggression and affective empathy (Giancola 2003). As such, it was difficult to determine the extent to which the present dosages of alcohol affected executive functioning and the subsequent effects of this on moral decision-making. Future research should consider exploring the acute effects of alcohol in both social processing and executive function-based tasks.

Secondly, the order of tasks following the intervention may influence results. The moral judgment and moral action tasks preceded the post-intervention affective empathy tasks. This decision was made to ensure that moral judgment and moral action tasks were completed during the window of peak BAC level. However, it might be that completing moral decision-making tasks first subsequently influenced the outcome in the empathy tasks. Consequently, future research should consider counterbalancing the order of these tasks to control for possible carry-over effects.

Importantly, the design of the present experiment, in terms of sample size and alcohol dosing, was based on previous research (Duke and Begue 2015). Whilst the average peak BAC levels recorded here were similar to the average recorded by Duke and Begue (2015), the range of BAC levels was smaller in the present investigation as a result of incorporating controlled dosages. Future research might consider incorporating higher dosages of alcohol that would extend these findings to the upper limits of the BAC level ranges reported in Duke and Begue’s field studies (0.05–0.16%). It is also important to note that sample sizes in the present study were based on Duke and Begue’s field studies and given that this investigation extends these original field studies by including additional measures in a mixed model experimental design, power may be affected. Future research should consider extending the present work to larger and more diverse sample populations. Further, given that previous research has reported mixed findings regarding gender differences in ethanol metabolic rates and subsequent blood alcohol levels (e.g. Frezza et al. 1990; Thomasson 2002) and that there has been evidence supporting gender differences in subjective ratings of facial displays of emotion (e.g. Lang et al. 1993; Montagne et al. 2005) and neural mechanisms underlying the processing of the pain of others (e.g. Han et al. 2008), future research should investigate the interaction effects of gender differences on acute alcohol effects on affective empathy and moral decision-making. Gender effects were not initially examined here as the gender composition of each group did not allow such a comparative analysis (however following suggestions from an anonymous reviewer, see Supplementary Material for post hoc analyses controlling for gender).

Following from these methodological considerations, it is also important to consider the roles of dispositional traits and behavioural states in the current discussion. Dispositional traits are thought to reflect core personality profiles (e.g. Haslam et al. 2004), with moral traits playing an important role in shaping our personal identity (Strohminger and Nichols 2014). Arguably and following from this, it is unlikely that the small to moderate dosages of alcohol in the present experiment would alter core moral principles shaped by social and moral norms, despite influencing behavioural and state-dependent measures of affective empathy and social processing (Duke and Begue 2015). Investigations should advance beyond the manipulation of state-dependent empathic processing and investigate moral judgments and simulated moral actions in populations in which there are likely to be distinct dispositional trait profiles. It is also important to highlight a limitation of incorporating relative measures of moral judgment, which assert that utilitarian and non-utilitarian (or deontological) motivations operate inversely (Patil 2015). Process dissociation approaches have revealed that moral ideologies guide moral judgments independently (Conway and Gawronski 2013; Conway et al. 2018) and as such, relative measures may fail to detect both utilitarian and deontological inclinations. Whether this process dissociation translates to moral actions requires further investigation but future work should expand these investigations to examine whether alcohol consumption, and subsequent changes in social processing, results in varying levels of both utilitarian and deontological inclinations in moral decision-making.

## Conclusion

Given the theory that increased utilitarian endorsements are driven by diminished affective empathy (e.g. Duke and Begue 2015) and aversion to harm (e.g. Patil 2015), we examined these components specifically in the present experiment, revealing a complex picture. Consuming alcohol at higher dosages did reduce affective empathy in a facial responding task, but this did not alter moral decision-making; moral actions continued to be dominated by utilitarian responses and moral judgments primarily comprised non-utilitarian responses. Given that the sample used in this investigation was predominately female and sampled from a student population, we do not attempt to make broad generalisations given sample representativeness. The outcomes of the present investigation might suggest that facets beyond or in addition to deficits in affective empathy influence the relationship between alcohol consumption and utilitarian endorsements (Duke and Begue 2015) and psychopathic traits and utilitarian endorsements (e.g. Francis et al. 2016; Francis et al. 2017b). Future research should consider expanding the present investigation in order to determine if these effects generalise more broadly.

## Electronic supplementary material

ESM 1 (DOCX 31.2 kb)

## Footnotes

1 Given that ‘empathy’ encompasses a variety of related phenomena (Decety and Cowell 2015), here we investigate the affective components of empathy specifically and define affective empathy as ‘...the generation of an appropriate emotional reaction in response to others’ emotions’ (Feshbach, 1978; 1987 as adopted by and cited in Wai and Tiliopoulos 2012).

2 The psychopharmacological effects of alcohol consumption are not reviewed in length here as alcohol has several mechanisms of action on the central nervous system (Carlson 2010); alcohol produces a complex effect that is mediated by distinct receptor systems that are not evenly amplified with ethanol dosage (Stoleman 2010). For example, among other effects, alcohol facilitates GABA transmission, inhibits glutamatergic transmission, and increases serotonin transmission (Stoleman 2010). Each of these prompts and mediates various anxiolytic, sedative, stimulant, and reinforcing effects (Carlson 2010).

3 Self-reported valence towards happy faces was negatively predicted by psychopathy, with valence towards sad faces showing the inverse effect (Ali et al. 2009; Wai and Tiliopoulos 2012).

4 Given their focus on the welfare of others, these traits are also inversely associated with primary psychopathy (Glenn et al. 2010; Lee and Ashton 2014).

5 The HEXACO scale was minimised to the Honesty-Humility (*α* = .79) and Agreeableness (*α* = .82) facets. Agreeableness was also included in the present investigation given its inverse association with anger and aggression (Ashton and Lee 2007); states also associated with alcohol consumption (Giancola 2004).

6 See Francis et al. (2017a) supporting information for validation studies; two experiments with qualitatively different populations found no differences in responses to the *footbridge* dilemma and the matched dilemma.

7 Whilst previous research has examined action-choice questions (behavioural question) as distinct from judgment questions (morality question), both of these remain self-reported moral judgments with a behavioural question only being ‘…what the participants *think* their action *could be* if they were to make the decision in real life’ (Tassy et al. 2013b, p. 2). Whereas, virtual reality offers the opportunity to present dynamic environments in which researchers can investigate theoretical and normative decisions in the framework of moral action (Navarrete et al. 2012) in which we can ask whether someone would ‘…actually resort to this course of action when the full repertoire of contextual features comes into play?’ (Patil et al. 2014, p. 95).

8 Although previous research has investigated arousal as a predictor of moral decisions in virtual dilemmas (Navarrete et al. 2012; Patil et al. 2014), in the present paradigm, heart rate change also incorporates the time in which participants witness the consequences of their actions. Given that we measure arousal beyond decision-making, we do not investigate predictions of moral actions from heart rate change in the present experiment (Francis et al. 2016).

9 This questionnaire also assessed exclusion criteria for the present experiment including alcohol naivety, alcohol dependence (including that of family members), and current medication use. These criteria had been outlined to participants prior to signing up to the experiment but were also included during the experiment as a precautionary measure.

10 Peak BAC was estimated using previous research (Mitchell Jr. et al. 2014) and initial pilot testing; participants (*N* = 7, *M*_age_ = 27.72, 3 females, 4 males) who consumed alcohol at low and high dosage reached peak BAC between approximately 20–40 min. All participants receiving the low or high alcohol dosages reached the target BAC levels in this time frame (low, 0.01%; high, 0.04%).

11 In order to determine if there were differences in responses to questions, an additional GEE analysis incorporating both the morality and behavioural questions revealed a main effect of task (Wald *X*^2^[1] = 36.28, *p* < .001), with a greater proportion of utilitarian responses overall in the action task as compared with both questions in the judgment task (*p*s < .001) but no difference between moral responses to the morality question and behavioural question (*p* = 1.00). There was no main effect of condition (*p* = .229) and no interaction (*p* = .466).

## References

[CR1] Ali F, Amorim IS, Chamorro-Premuzic T (2009). Empathy deficits and trait emotional intelligence in psychopathy and Machiavellianism. Personal Individ Differ.

[CR2] Aquino K, Reed A (2002). The self-importance of moral identity. J Pers Soc Psychol.

[CR3] Ashton MC, Lee K (2007). Empirical, theoretical, and practical advantages of the HEXACO model of personality structure. Personal Soc Psychol Rev.

[CR4] Ashton MC, Lee K (2009). The HEXACO-60: a short measure of the major dimensions of personality. J Pers Assess.

[CR5] Ashton MC, Lee K, de Vries RE (2014). The HEXACO honesty-humility, agreeableness, and emotionality factors: a review of research and theory. Personal Soc Psychol Rev.

[CR6] Assefi SL, Garry M (2003). Absolut memory distortions: alcohol placebos influence the misinformation effect. Psychol Sci.

[CR7] Bartels DM, Pizarro DA (2011). The mismeasure of morals: antisocial personality traits predict utilitarian responses to moral dilemmas. Cognition.

[CR8] Bartels DM, Bauman CW, Cushman FA, Pizarro DA, McGraw AP, Keren G, Wu G (2015). Moral judgment and decision making. The Wiley Blackwell handbook of judgment and decision making.

[CR9] Batson C. Daniel (2009). These Things Called Empathy: Eight Related but Distinct Phenomena. The Social Neuroscience of Empathy.

[CR10] Bauman CW, McGraw AP, Bartels DM, Warren C (2014). Revisiting external validity: concerns about trolley problems and other sacrificial dilemmas in moral psychology. Soc Personal Psychol Compass.

[CR11] Beaupré, M., Cheung, N., & Hess, U. (2000). The Montreal set of facial displays of emotion [Slides]. Available from Ursula Hess, Department of Psychology, University of Quebec at Montreal, PO Box, 8888

[CR12] Blair RJ (1995). A cognitive developmental approach to mortality: investigating the psychopath. Cognition.

[CR13] Bradley MM, Lang PJ (1994). Measuring emotion: the self-assessment manikin and the semantic differential. J Behav Ther Exp Psychiatry.

[CR14] Brick C, Lewis GJ (2016). Unearthing the “green” personality: core traits predict environmentally friendly behavior. Environ Behav.

[CR15] Carlson NR (2010). Physiology of behaviour (Tenth ed.).

[CR16] Carmona-Perera M, Reyes Del Paso GA, Perez-Garcia M, Verdejo-Garcia A (2013). Heart rate correlates of utilitarian moral decision-making in alcoholism. Drug Alcohol Depend.

[CR17] Carmona-Perera M, Clark L, Young L, Perez-Garcia M, Verdejo-Garcia A (2014). Impaired decoding of fear and disgust predicts utilitarian moral judgment in alcohol-dependent individuals. Alcohol Clin Exp Res.

[CR18] Carney DR, Mason MF (2010). Decision making and testosterone: when the ends justify the means. J Exp Soc Psychol.

[CR19] Choe SY, Min KH (2011). Who makes utilitarian judgments? The influences of emotions on utilitarian judgments. Judgm Decis Mak.

[CR20] Ciaramelli E, Muccioli M, Ladavas E, di Pellegrino G (2007). Selective deficit in personal moral judgment following damage to ventromedial prefrontal cortex. Soc Cogn Affect Neurosci.

[CR21] Cima M, Tonnaer F, Hauser M (2010). Psychopaths know right from wrong but don’t care. Soc Cogn Affect Neurosci.

[CR22] Cohen TR, Panter AT, Miller CB, Furr RM, Knobel A, Fleeson W (2015). Character traits in the workplace: a three-month diary study of moral and immoral organizational behaviors. Character: new directions from philosophy, psychology, and theology.

[CR23] Cohen TR, Panter AT, Turan N, Morse L, Kim Y (2014). Moral character in the workplace. J Pers Soc Psychol.

[CR24] Conway P, Gawronski B (2013). Deontological and utilitarian inclinations in moral decision making: a process dissociation approach. J Pers Soc Psychol.

[CR25] Conway P, Goldstein-Greenwood J, Polacek D, Greene JD (2018). Sacrificial utilitarian judgments do reflect concern for the greater good: clarification via process dissociation and the judgments of philosophers. Cognition.

[CR26] Crockett MJ, Clark L, Hauser M, Robbins TW (2010). Serotonin selectively influences moral judgment and behavior through effects on harm aversion. Proc Natl Acad Sci.

[CR27] Cushman F, Young L, Greene J, Doris JM (2010). Our multi-system moral psychology: towards a consensus view. The moral psychology handbook.

[CR28] Davis MH (1983). Measuring individual-differences in empathy - evidence for a multidimensional approach. J Pers Soc Psychol.

[CR29] Decety J, Cowell JM (2015). Empathy, justice, and moral behavior. AJOB Neurosci.

[CR30] Decety J, Jackson PL (2004). The functional architecture of human empathy. Behav Cogn Neurosci Rev.

[CR31] Decety J, Moriguchi Y (2007). The empathic brain and its dysfunction in psychiatric populations: implications for intervention across different clinical conditions. Biopsychosocial Medicine.

[CR32] Djeriouat H, Tremoliere B (2014). The dark triad of personality and utilitarian moral judgment: the mediating role of honesty/humility and harm/care. Personal Individ Differ.

[CR33] Duke AA, Begue L (2015). The drunk utilitarian: blood alcohol concentration predicts utilitarian responses in moral dilemmas. Cognition.

[CR34] Ferrari V, Smeraldi E, Bottero G, Politi E (2014). Addiction and empathy: a preliminary analysis. Neurol Sci.

[CR35] Foot P (1978). Virtues and vices and other essays in moral philosophy.

[CR36] Francis KB, Howard C, Howard I, Gummerum M, Ganis G, Anderson G, Terbeck S (2016). Virtual morality: transitioning from moral judgment to moral action?. PLoS One.

[CR37] Francis KB, Gummerum M, Ganis G, Howard IS, Terbeck S (2017). Virtual morality in the helping professions: simulated action and resilience. Br J Psychol.

[CR38] Francis KB, Terbeck S, Briazu RA, Haines A, Gummerum M, Ganis G, Howard IS (2017). Simulating moral actions: an investigation of personal force in virtual moral dilemmas. Sci Rep.

[CR39] Frezza Mario, di Padova Carlo, Pozzato Gabriele, Terpin Maddalena, Baraona Enrique, Lieber Charles S. (1990). High Blood Alcohol Levels in Women. New England Journal of Medicine.

[CR40] Gao Y, Tang S (2013). Psychopathic personality and utilitarian moral judgment in college students. J Crim Just.

[CR41] Giancola PR (2000). Executive functioning: a conceptual framework for alcohol-related aggression. Exp Clin Psychopharmacol.

[CR42] Giancola PR (2003). The moderating effects of dispositional empathy on alcohol-related aggression in men and women. J Abnorm Psychol.

[CR43] Giancola PR (2004). Executive functioning and alcohol-related aggression. J Abnorm Psychol.

[CR44] Glenn AL, Iyer R, Graham J, Koleva S, Haidt J (2009). Are all types of morality compromised in psychopathy?. J Personal Disord.

[CR45] Glenn AL, Koleva S, Iyer R, Graham J, Ditto PH (2010). Moral identity in psychopathy. Judgm Decis Mak.

[CR46] Godlaski AJ, Giancola PR (2009). Executive functioning, irritability, and alcohol-related aggression. Psychol Addict Behav.

[CR47] Gold N, Pulford BD, Colman AM (2015). Do as I say, don’t do as I do: differences in moral judgments do not translate into differences in decisions in real-life trolley problems. J Econ Psychol.

[CR48] Greene JD, Sommerville RB, Nystrom LE, Darley JM, Cohen JD (2001). An fMRI investigation of emotional engagement in moral judgment. Science.

[CR49] Greene JD, Nystrom LE, Engell AD, Darley JM, Cohen JD (2004). The neural bases of cognitive conflict and control in moral judgment. Neuron.

[CR50] Greene JD, Morelli SA, Lowenberg K, Nystrom LE, Cohen JD (2008). Cognitive load selectively interferes with utilitarian moral judgment. Cognition.

[CR51] Han Shihui, Fan Yan, Mao Lihua (2008). Gender difference in empathy for pain: An electrophysiological investigation. Brain Research.

[CR52] Haslam N, Bastian B, Bissett M (2004). Essentialist beliefs about personality and their implications. Personal Soc Psychol Bull.

[CR53] Hu Y, Cui Z, Fan M, Pei Y, Wang Z (2017). Effects of acute alcohol intoxication on empathic neural responses for pain. Front Hum Neurosci.

[CR54] Jackson PL, Meltzoff AN, Decety J (2005). How do we perceive the pain of others? A window into the neural processes involved in empathy. NeuroImage.

[CR55] Kahane G, Everett JA, Earp BD, Farias M, Savulescu J (2015). ‘Utilitarian’ judgments in sacrificial moral dilemmas do not reflect impartial concern for the greater good. Cognition.

[CR56] Khemiri L, Guterstam J, Franck J, Jayaram-Lindstrom N (2012). Alcohol dependence associated with increased utilitarian moral judgment: a case control study. PLoS One.

[CR57] Koenigs M, Young L, Adolphs R, Tranel D, Cushman F, Hauser M, Damasio A (2007). Damage to the prefrontal cortex increases utilitarian moral judgements. Nature.

[CR58] Koenigs M, Kruepke M, Zeier J, Newman JP (2012). Utilitarian moral judgment in psychopathy. Soc Cogn Affect Neurosci.

[CR59] LANG PETER J., GREENWALD MARK K., BRADLEY MARGARET M., HAMM ALFONS O. (1993). Looking at pictures: Affective, facial, visceral, and behavioral reactions. Psychophysiology.

[CR60] Lee K, Ashton MC (2014). The dark triad, the big five, and the HEXACO model. Personal Individ Differ.

[CR61] Levenson MR, Kiehl KA, Fitzpatrick CM (1995). Assessing psychopathic attributes in a noninstitutionalized population. J Pers Soc Psychol.

[CR62] Marinkovic K, Oscar-Berman M, Urban T, O’Reilly CE, Howard JA, Sawyer K, Harris GJ (2009). Alcoholism and dampened temporal limbic activation to emotional faces. Alcohol Clin Exp Res.

[CR63] Maurage P, Grynberg D, Noël X, Joassin F, Philippot P, Hanak C, Verbanck P, Luminet O, de Timary P, Campanella S (2011). Dissociation between affective and cognitive empathy in alcoholism: a specific deficit for the emotional dimension. Alcohol Clin Exp Res.

[CR64] McDonald MM, Defever AM, Navarrete CD (2017). Killing for the greater good: action aversion and the emotional inhibition of harm in moral dilemmas. Evol Hum Behav.

[CR65] Mendez MF, Anderson E, Shapira JS (2005). An investigation of moral judgement in frontotemporal dementia. Cogn Behav Neurol.

[CR66] Miller JD, Gaughan ET, Pryor LR (2008). The Levenson Self-Report Psychopathy scale: an examination of the personality traits and disorders associated with the LSRP factors. Assessment.

[CR67] Mitchell MC, Teigen EL, Ramchandani VA (2014). Absorption and peak blood alcohol concentration after drinking beer, wine, or spirits. Alcohol Clin Exp Res.

[CR68] Montagne Barbara, Kessels Roy P. C., Frigerio Elisa, de Haan Edward H. F., Perrett David I. (2005). Sex differences in the perception of affective facial expressions: Do men really lack emotional sensitivity?. Cognitive Processing.

[CR69] Navarrete CD, McDonald MM, Mott ML, Asher B (2012). Virtual morality: emotion and action in a simulated three-dimensional “trolley problem”. Emotion.

[CR70] Parsons TD (2015). Virtual reality for enhanced ecological validity and experimental control in the clinical, affective and social neurosciences. Front Hum Neurosci.

[CR71] Partala Timo, Surakka Veikko (2003). Pupil size variation as an indication of affective processing. International Journal of Human-Computer Studies.

[CR72] Patil I (2015). Trait psychopathy and utilitarian moral judgement: the mediating role of action aversion. J Cogn Psychol.

[CR73] Patil I, Cogoni C, Zangrando N, Chittaro L, Silani G (2014). Affective basis of judgment-behavior discrepancy in virtual experiences of moral dilemmas. Soc Neurosci.

[CR74] Perkins AM, Leonard AM, Weaver K, Dalton JA, Mehta MA, Kumari V, Williams SCR, Ettinger U (2013). A dose of ruthlessness: interpersonal moral judgment is hardened by the anti-anxiety drug lorazepam. J Exp Psychol Gen.

[CR75] Roberts W, Fillmore MT, Milich R (2012). Drinking to distraction: does alcohol increase attentional bias in adults with ADHD?. Exp Clin Psychopharmacol.

[CR76] Schmitt WA, Newman JP (1999). Are all psychopathic individuals low-anxious?. J Abnorm Psychol.

[CR77] Stoleman IP (2010). Encyclopedia of psychopharmacology.

[CR78] Strohminger N, Nichols S (2014). The essential moral self. Cognition.

[CR79] Tassy S, Deruelle C, Mancini J, Leistedt S, Wicker B (2013). High levels of psychopathic traits alters moral choice but not moral judgment. Front Hum Neurosci.

[CR80] Tassy S, Oullier O, Mancini J, Wicker B (2013). Discrepancies between judgment and choice of action in moral dilemmas. Front Psychol.

[CR81] Thoma P, Friedmann C, Suchan B (2013). Empathy and social problem solving in alcohol dependence, mood disorders and selected personality disorders. Neurosci Biobehav Rev.

[CR82] Thomasson Holly R. (2002). Gender Differences in Alcohol Metabolism. Recent Developments in Alcoholism.

[CR83] Thomson JJ (1976). Killing, letting die, and the trolley problem. Monist.

[CR84] Visser BA, Ashton MC, Pozzebon JA (2012). Is low anxiety part of the psychopathy construct?. J Pers.

[CR85] Wai M, Tiliopoulos N (2012). The affective and cognitive empathic nature of the dark triad of personality. Personal Individ Differ.

[CR86] Weller JA, Tikir A (2011). Predicting domain-specific risk taking with the HEXACO personality structure. J Behav Decis Mak.

