# Supplementary material for: Alcohol, empathy, and morality: acute effects of alcohol consumption on affective empathy and moral decision-making
Source: Psychopharmacology (Berl). 2019 Jul 9;236(12):3477–96. doi: 10.1007/s00213-019-05314-z (PMC6892760; doi:10.1007/s00213-019-05314-z)
Supplement: Supplementary file 1 — (DOCX 31.2 kb) [file 213_2019_5314_MOESM1_ESM.docx]

*Psychopharmacology*

Alcohol, Empathy, and Morality:
Acute effects of alcohol consumption on affective empathy and moral decision-making

Kathryn B. Francis ^1,3*^, Michaela Gummerum^1^, Giorgio Ganis^1^, Ian S. Howard^2^, & Sylvia Terbeck^1^.

^1^University of Plymouth, School of Psychology, Drake Circus, Plymouth, PL4 8AA, United Kingdom

^2^University of Plymouth, Centre for Robotics and Neural Systems, Drake Circus, Plymouth, PL4 8AA, United Kingdom

^3^Present affiliation: Division of Psychology, Faculty of Management, Law & Social Sciences, University of Bradford, Bradford, West Yorkshire, BD7 1DP, United Kingdom.

* Corresponding author

E-mail: kathrynbfrancis@googlemail.com

**Supplementary Material**

**Control Variables and Checks**

**Order effects.** Across groups, no order effects were found based on task presentation when referencing the morality question (*p* = .437) or behavioural question (*p* = .775) in GEE with order effect (action first; action last) and task (judgment; action) as within-subjects variables. Additionally, as in previous research (see Francis et al., 2016), endorsing a utilitarian outcome in the moral action task (pushing the person in virtual reality) was not associated with prior gaming experience (*ps* > .381) in point-biserial correlational analyses.

**BAC level.** In order to determine if the low and high alcohol interventions had increased BAC levels, a comparison between peak BAC levels (20 minutes after alcohol consumption) was first completed. Average peak BAC levels (%) were highest in the high alcohol condition (*M* = 0.03%, *SD* = 0.01, *Range* = 0.01% - 0.05%), moderately high in the low alcohol condition (*M* = 0.01%, *SD* = 0.01, *Range* = 0% - 0.03%), and as expected, were absent in the placebo condition. A one-way ANOVA was performed with condition entered as the between-subjects variable (placebo; low alcohol; high alcohol) and peak BAC level entered as the dependent variable. Analysis found a significant difference in peak BAC levels between conditions, (*F*(2, 45) = 51.97, *p* < .001) with follow-up tests revealing significant differences between the placebo and low alcohol conditions, (*p* = .005, *d* = -1.66), the placebo and high alcohol conditions, (*p* < .001, *d* = -3.58), and the low and high alcohol conditions, (*p* < .001, *d* = -1.92).

**Awareness check.** Awareness checks revealed that 87.5% of participants in the placebo condition were unaware of condition assignment and did not know whether they had consumed alcohol or not. In the low alcohol condition, 68.75% of participants reported that they did not know whether they had consumed alcohol or not and this decreased to 31.25% in the high alcohol condition. However, reported awareness of condition assignment was not associated with either moral actions (*p* = .521) or moral judgments (when referencing either the action-choice and judgment question) (*ps* > .388) when controlling for condition in Fisher’s exact tests. As such, awareness checks were not included in further analyses.

**Drinking habits.** Having measured drinking habits (total units of alcohol consumed weekly), preliminary analyses were performed to identify possible confounding effects of alcohol tolerance on condition assignment and BAC level. A one-way ANOVA with condition (placebo; low alcohol; high alcohol) as the between-subjects variable and drinking habits as the dependent variable, found no significant differences between the total units of alcohol consumed weekly between conditions (*p* = .328). Further, self-reported total units of alcohol consumed weekly did not correlate with peak BAC level (*p* = .975). As such, drinking habits were not included in further analyses.

**Subjective mood.** With previous research suggesting that the relationship between alcohol and moral decision-making may be influenced by feelings of disinhibition or positive affect (Duke & Begue, 2015), subjective mood ratings (disinhibition; positive affect) were compared before and after the alcohol intervention. A two-way mixed model ANOVA with disinhibition as the dependent variable, condition (placebo; low alcohol; high alcohol) as the between-subjects factor and time completed (pre-intervention; post-intervention) as the within-subjects variable, revealed no effect of condition (*p* = .740) or time completed (*p* = .938) or subsequent interaction effects (*p* = .096) on subjective reports of disinhibition. Therefore, disinhibition was not included in further analysis. A second ANOVA with positive affect entered in the model as the dependent variable, revealed a main effect of time on subjective reports of happiness, (*F*(1, 45) = 9.18, *p* = .004, *d* = 0.32) with subjective reports of happiness significantly lower after the intervention. However, there were no main effects of condition (*p* = .804) and no interaction effects (*p* = .850). Point-biserial correlations revealed no relationship between subjective ratings of positive affect following the intervention and moral actions (*p* = .673) or moral judgments (when referencing either the judgment or action-choice question) (*ps* > .175). As such, positive affect was not included in further analysis.

**Heart Rate and Alcohol**

Table I
*Mean Heart Rate Change (bpm) across Judgment and Action Tasks*

|  | Judgment task | | |  | Action task | | |
| --- | --- | --- | --- | --- | --- | --- | --- |
| Condition | Non-moral |  | Moral |  | Non-moral |  | Moral |
|  | *M (SD)* |  | *M (SD)* |  | *M (SD)* |  | *M (SD)* |
| Placebo | -0.63 (0.81) |  | 0.06 (1.18) |  | -0.25 (2.02) |  | 2.81 (3.35) |
| Low Alcohol | -0.75 (0.93) |  | -0.13 (1.59) |  | -0.50 (2.37) |  | 2.13 (3.34) |
| High Alcohol | -0.88 (0.72) |  | -0.06 (1.39) |  | -0.06 (1.06) |  | 0.94 (3.59) |

**Alcohol and Behavioural Measures of Affective Empathy**

Table II
*Average valence scores (1 (negative) – 9 (positive)) across each emotion set of facial expressions (neutral; happy; sad) for the pre-intervention and post-intervention tests across conditions.*

|  | Pre-intervention | | | | |  | Post-intervention | | | | |
| --- | --- | --- | --- | --- | --- | --- | --- | --- | --- | --- | --- |
| Condition | Neutral |  | Happy |  | Sad |  | Neutral |  | Happy |  | Sad |
|  | *M (SD)* |  | *M (SD)* |  | *M (SD)* |  | *M (SD)* |  | *M (SD)* |  | *M (SD)* |
| Placebo | 4.72 (0.34) |  | 6.80 (1.01) |  | 3.45 (0.83) |  | 4.81 (0.38) |  | 6.72 (1.12) |  | 3.42 (1.02) |
| Low Alcohol | 4.56 (0.69) |  | 6.49 (0.91) |  | 3.63 (0.89) |  | 4.75 (0.42) |  | 6.67 (0.85) |  | 3.72 (1.05) |
| High Alcohol | 4.70 (0.60) |  | 7.02 (1.08) |  | 3.10 (1.09) |  | 4.83 (0.71) |  | 6.39 (1.10) |  | 3.77 (1.39) |

Table III
*Average empathy for pain scores (0 (no pain) - 10 (worse pain ever))* *for neutral and painful images for the pre-intervention and post-intervention tests across conditions.*

|  | Pre-Intervention | | |  | Post-Intervention | | |
| --- | --- | --- | --- | --- | --- | --- | --- |
| Condition | Neutral |  | Painful |  | Neutral |  | Painful |
|  | *M (SD)* |  | *M (SD)* |  | *M (SD)* |  | *M (SD)* |
| Placebo | 0.75 (0.64) |  | 6.89 (1.17) |  | 0.26 (0.42) |  | 7.16 (1.35) |
| Low Alcohol | 1.03 (1.16) |  | 7.01 (1.08) |  | 0.61 (0.90) |  | 7.19 (1.40) |
| High Alcohol | 1.00 (0.91) |  | 6.89 (1.65) |  | 0.62 (0.86) |  | 6.76 (1.89) |

Table IV
*Correlation between valence scores for facial emotions in the SAM facial test and trait measures*

| Trait measure | Subscale |  | Facial test (SAM)^a^ | | |
| --- | --- | --- | --- | --- | --- |
|  |  |  | Valence | | |
|  |  |  | Neutral (*r*) | Happy (*r*) | Sad (*r*) |
| 1. LPS |  |  |  |  |  |
|  | Primary |  | .13 | -.34* | .39** |
|  | Secondary |  | -.09 | -.01 | .04 |
| 1. HEXACO |  |  |  |  |  |
|  | H |  | -.13 | .35* | -.47** |
|  | A |  | .26 | .11 | -.08 |
| 1. IRI |  |  |  |  |  |
|  | PT |  | .08 | .05 | -.19 |
|  | EC |  | -.14 | .15 | -.30* |
|  | PD |  | -.05 | -.14 | .06 |
|  | FS |  | .08 | .13 | .02 |
| 1. Moral Identity |  |  |  |  |  |
|  | Symbolization |  | -.12 | .26 | -.18 |
|  | Internalization |  | -.18 | .29* | -.27 |

*Note.* *r* = correlation coefficient; H = Honesty-Humility; A = Agreeableness; PT = Perspective Taking; EC = Empathic Concern; PD = Personal Distress; FS = Fantasy.
^a^Valence scores from the SAM facial pre-intervention test only.
**p* <.05. ***p* <.01. ****p* <.001

| Trait measure | Subscale |  | Empathy for pain^b^ | |
| --- | --- | --- | --- | --- |
|  |  |  | VAS score | |
|  |  |  | Neutral | Painful |
| 1. LPS |  |  |  |  |
|  | Primary |  | -.24 | -.35* |
|  | Secondary |  | -.06 | .11 |
| 1. HEXACO |  |  |  |  |
|  | H |  | .32* | .21 |
|  | A |  | .12 | -.08 |
| 1. IRI |  |  |  |  |
|  | PT |  | .05 | -.00 |
|  | EC |  | .09 | .31* |
|  | PD |  | -.08 | .15 |
|  | FS |  | -.21 | -.03 |
| 1. Moral Identity |  |  |  |  |
|  | Symbolization |  | .18 | .04 |
|  | Internalization |  | .11 | .27 |

Table V
*Correlations between VAS scores in the pain test and trait measures*

*Note.* H = Honesty-Humility; A = Agreeableness; PT = Perspective Taking; EC = Empathic Concern; PD = Personal Distress; FS = Fantasy.
^a^VAS scores from the empathy for pain pre-intervention test only.
**p* <.05. ***p* <.01. ****p* <.001

**Analyses: Controlling for Gender**

**Alcohol.** Previous research has reported mixed findings regarding gender differences in ethanol metabolic rates and subsequent blood alcohol levels (e.g., Frezza et al., 1990; Thomasson, 1995). In order to control for potential differences in BAC levels, an additional analysis was performed comparing BAC levels between conditions with gender as an additional between-subjects factor. Analysis revealed no main effect of gender on BAC level (*p* = .151) and no interaction between gender x condition (*p* = .331). Supporting previous analysis, a significant main effect of condition was found, (*F*(2, 42) = 42.14, *p* < .001).

**Behavioural empathy measures and alcohol.** There has been evidence supporting gender differences in subjective ratings of facial displays of emotion (e.g., Lang, Greenwald, Bradley, & Hamm, 1993; Montagne, Kessels, Frigerio, de Haan, & Perrett, 2005) and neural mechanisms underlying the processing of the pain of others (e.g., Han, Fan, & Mao, 2008). However, these findings have been mixed with research arguing that subjective reports are often “contaminated by…a bias to confirm the sex-role stereotypes” (Han et al., 2008, p. 86).

To control for potential interaction effects of gender differences in subjective ratings of facial displays of emotion, analysis examining differences in self-reported valence between conditions was repeated with gender entered as a dummy-coded covariate (male; female). Analysis revealed that existing effects and interactions remained significant when controlling for gender.

To control for potential interaction effects of gender differences in subjective ratings of the pain of others, analysis examining differences in empathy for pain scores between conditions was repeated with gender entered as a dummy-coded covariate (male; female). Analysis revealed that the existing main effect of image type remained. The marginally significant main effect of test and significant interaction of test x image type were no longer significant following the inclusion of gender (*ps* > .412). Follow-up analyses revealed a marginally significant interaction of test x image type x gender (*p* = .064) with females giving lower VAS scores for neutral images in the post-test compared to the pre-test (*p* < .001). However, there were significant violations of homogeneity of variances, likely driven by the difference in the ratio of females (*N* = 31) to males (*N* = 17). As such, these analyses must be treated with caution and further research is required before interpreting true gender differences.

**Behavioural empathy measures and traits.** Partial correlations were performed between pre-intervention valence scores and traits while controlling for gender. Analysis revealed that all the existing associations remained (original results reported in green):

Individuals scoring higher in primary psychopathy felt more positively when looking at sad facial expressions, (*r*(46) = .36, *p* =.012) (*r*(46) = .39, *p* =.007) and more negatively when looking at happy facial expressions, (*r*(46) = -.34, *p* =.021) (*r*(46) = -.34, *p* =.017). Honesty-Humility, on the other hand, correlated negatively with valence towards sad facial expressions, (*r*(46) = -.44, *p* =.002) (*r*(46) = -.47, *p* =.001) and positively with valence towards happy facial expressions, (*r*(46) = .34, *p* =.019) (*r*(46) = .35, *p* =.015).

The findings regarding Internalization and Empathic Concern approached significance (these changes in significance were likely due to loss of power). Individuals scoring higher in Internalization, felt more positively towards happy facial expressions, (*r*(46) = .28, *p* =.055) (*r*(46) = .29, *p* =.048) and individuals with higher Empathic Concern scores, reported feeling more negative towards sad facial expressions, (*r*(46) = -.29, *p* =.051) (*r*(46) = -.30, *p* =.037).

Partial correlations were also performed between pre-intervention pain scores and traits while controlling for gender. Analysis revealed that all existing associations remained apart from the association between Honesty-Humility and greater empathy for pain scores when looking at neutral images (again, this is likely due to loss of power and this does not have substantial theoretical implications given that it concerns neutral images):

Individuals scoring higher in primary psychopathy had lower empathy for pain scores when looking at painful images, (*r*(46) = -.32, *p* =.029) (*r*(46) = -.35, *p* =.016). Empathic Concern, on the other hand, correlated positively with empathy for pain scores on the VAS in response to painful images, (*r*(46) = .30, *p* =.043) (*r*(46) = .31, *p* =.03). The finding that individuals scoring higher in Honesty-Humility reported greater empathy for pain scores when looking at neutral images, (*r*(46) = .32, *p* =.025) was not found when controlling for gender in the partial correlational analysis (*p* =.112).

**Behavioural empathy measures and moral responses.** Partial correlations were performed between changes in self-reported valence towards happy and sad faces between the pre- and post-intervention tests and moral responses (action; judgment) while controlling for gender. Results were unaffected. Changes in self-reported valence towards happy and sad faces between the pre- and post-intervention tests, were not associated with moral actions (*ps* > .486) or moral judgments (*ps* > .407) when controlling for gender.

Partial correlations were also performed between changes in empathy for pain scores towards neutral and painful images between the pre- and post-intervention tests and moral responses (action; judgment) while controlling for gender. Results were unaffected. Changes in empathy for pain scores between the pre- and post-interventions for neutral and painful images were not associated with either moral actions (*ps* > .203) or moral judgments (*ps* > .406).

Given the imbalanced ratio of males to females in the present sample, and the fact that sample size collection (and power analyses) did not account for gender comparisons (as these were completed post-hoc), interpreting true gender differences requires a larger, balanced sample.
